# Novel SNPs Linked to Blast Resistance Genes Identified in Pearl Millet Through Genome-Wide Association Models

**DOI:** 10.3390/ijms252212048

**Published:** 2024-11-09

**Authors:** Swati Singh, Ganesan Prakash, Sandeep Nanjundappa, Renuka Malipatil, Prerana Kalita, Tara C. Satyavathi, Nepolean Thirunavukkarasu

**Affiliations:** 1 Genomics and Molecular Breeding Lab, Global Centre of Excellence on Millets (Shree Anna), ICAR-Indian Institute of Millets Research, Hyderabad 500030, India; singh.swati1120@gmail.com (S.S.); sandeepgowda.n@gmail.com (S.N.); malipatilrenuka@gmail.com (R.M.); preranakalita007@gmail.com (P.K.); csatyavathi@gmail.com (T.C.S.); 2 Division of Plant Pathology, ICAR-Indian Agricultural Research Institute, New Delhi 110012, India; prakashg@iari.res.in

**Keywords:** pearl millet, blast, resistance genes, GWAS, biotic stress, disease resistance

## Abstract

Foliar blast, caused by *Pyricularia grisea*, poses a major challenge to pearl millet (*Pennisetum glaucum* (L.) R. Br) production, leading to severe yield losses, particularly in rainfed ecologies. This study aimed to elucidate the genetic basis of blast resistance through a genome-wide association study (GWAS) involving 281 diverse pearl millet inbreds. GWAS panel was phenotyped for blast resistance against three distinct isolates of *P. grisea* collected from Delhi, Gujarat, and Rajasthan locations, revealing a significant variability with 16.7% of the inbreds showing high resistance. Bayesian information and linkage disequilibrium iteratively nested keyway (BLINK) and Multi-Locus Mixed Model (MLMM) models using transformed means identified 68 significant SNPs linked to resistance, with hotspots for resistance-related genes on chromosomes 1, 2, and 6. These regions harbor genes involved in defense mechanisms, including immune response, stress tolerance, signal transduction, transcription regulation, and pathogen defense. Genes, namely *14-3-3-like proteins RGA2*, *RGA4*, *hypersensitive-induced response proteins*, *NHL3*, *NBS-LRR*, *LRR-RLK*, *LRRNT_2,* and various transcription factors such as *AP2/ERF* and *WRKY*, played a crucial role in the stress-responsive pathways. Analyses of transporter proteins, redox processes, and structural proteins revealed additional mechanisms contributing to blast resistance. This study offers valuable insights into the complex genetic architecture of blast resistance in pearl millet, offering a solid foundation for marker-assisted breeding programs and gene-editing experiments.

## 1. Introduction

Pearl millet (*Pennisetum glaucum* (L.) R. Br.), a diploid C4 plant, is a crucial cereal crop for arid and semi-arid regions due to its exceptional drought tolerance and ability to thrive in poor soils. It is also nutritionally superior to major cereals such as rice, wheat, and maize, comprising higher levels of protein, iron, and zinc [1]. Pearl millet plays a vital role in food and fodder security for populations reliant on low-input farming systems [2,3,4]. Its gluten-free nature and low glycemic index further position it as a promising candidate for the expanding global market of functional foods [5,6,7]. However, despite developing high-yielding hybrids and open-pollinated varieties, pearl millet productivity remains vulnerable to various biotic and abiotic stresses. Among the most significant biotic threats is blast disease, caused by the fungus *Pyricularia grisea*, which has become endemic in all production regions [8,9].

Blast disease is characterized by grey lesions on leaves and stems, starting as small lesions that expand into necrotic patches, leading to significant damage to the crop, including chlorosis and eventual death [10,11]. Both forage and grains of pearl millet have faced severe blast outbreaks in recent years, transforming foliar blast from a minor to a major threat to pearl millet cultivation globally [12]. Effective management strategies are essential to prevent further decline in crop yield. Although fungicide applications have been employed to control the disease, their long-term effectiveness and sustainability are challenged. Breeding for genetic resistance is the most effective and environmentally friendly approach. However, the genetic basis of resistance to foliar blast in pearl millet remains unexplored.

Although earlier studies suggested that a single dominant gene may control resistance to foliar blast, recent studies indicate that resistance likely involves multiple genes, reflecting a more complex inheritance pattern [9,13,14,15]. The emergence of new pathotypes and ongoing host–pathogen interactions further suggest the involvement of multiple genes, underscoring the necessity for their identification [16]. The advent of high-throughput sequencing and the development of SNP genotyping platforms have made it increasingly feasible to study complex traits in crops [17]. Mid-density SNP genotyping platforms such as AgriSeq have been successfully used for SNP genotyping in pearl millet, enabling efficient and reproducible genomic studies [18,19].

Genome-wide association approach (GWAS) is a powerful tool [20,21] for identifying genetic loci associated with different traits, including disease resistance, across various crops [22,23]. This strategy facilitates the identification of multiple alleles at each locus, enabling the study of how different alleles affect blast resistance [24]. The GWAS approach has successfully identified genetic loci associated with blast resistance in various cereal crops. In rice, several loci involved in panicle blast resistance have been discovered, while in wheat, temperature-sensitive resistance genes, namely *Rmg2* and *Rmg3*, were identified [25,26]. In rice, two panicle blast resistance genes, *Pb2* and *Pb3*, were identified using GWAS on a diversity panel featuring 700,000 SNP markers [25,26,27]. Research on finger millet and foxtail millet has revealed genomic regions associated with blast resistance, providing insights into the shared and unique resistance mechanisms among cereals [28]. GWAS identified resistant loci, namely *seita.2G097500* and *seita.2G097400*, on chromosomes 2 and 9 for blast in foxtail millet, with eight putative genes linked to rice blast resistance pathways [28]. Despite the availability of genomic resources in pearl millet, GWAS has not been extensively used to investigate blast resistance.

Considering the economic losses caused by blast disease in various pearl millet production ecologies, our study aims to generate comprehensive phenotypic data from isolates of *P. grisea* found in prominent pearl millet production areas such as Rajasthan, Gujarat, and Delhi and to identify the significant SNPs and genes associated with blast resistance through GWAS. Our findings will offer valuable insights and molecular markers for use in breeding programs and gene-editing approaches, ultimately contributing to the development of blast-resistant pearl millet varieties and enhancing crop resilience in rain-fed ecologies.

## 2. Results

### 2.1. Phenotypic Variation for Blast Resistance in the GWAS Panel

The distribution of blast scores across the genotypes revealed considerable phenotypic variation. Among the 281 inbreds, 47 (16.7%) were classified as highly resistant (scores ≤ 1), and 6 (2.1%) were highly susceptible (scores of 8–9). The groups categorized as moderately resistant (scores of 2 to 3) and moderately susceptible (scores of 4 to 5) comprised 20.6% and 18.5% of the inbreds, respectively.

Analysis of variance among the genotypes screened for the different blast isolates revealed substantial variation in blast resistance (Table 1). Heritability estimates for disease resistance ranged from 86.4% to 93.8%, with the highest values observed in the pooled data (93.8%) and the lowest for PMg_Del (86.3%). These heritability estimates provide insights into the reliability of the phenotypic scores for subsequent genetic analyses.

**Figure 1:**
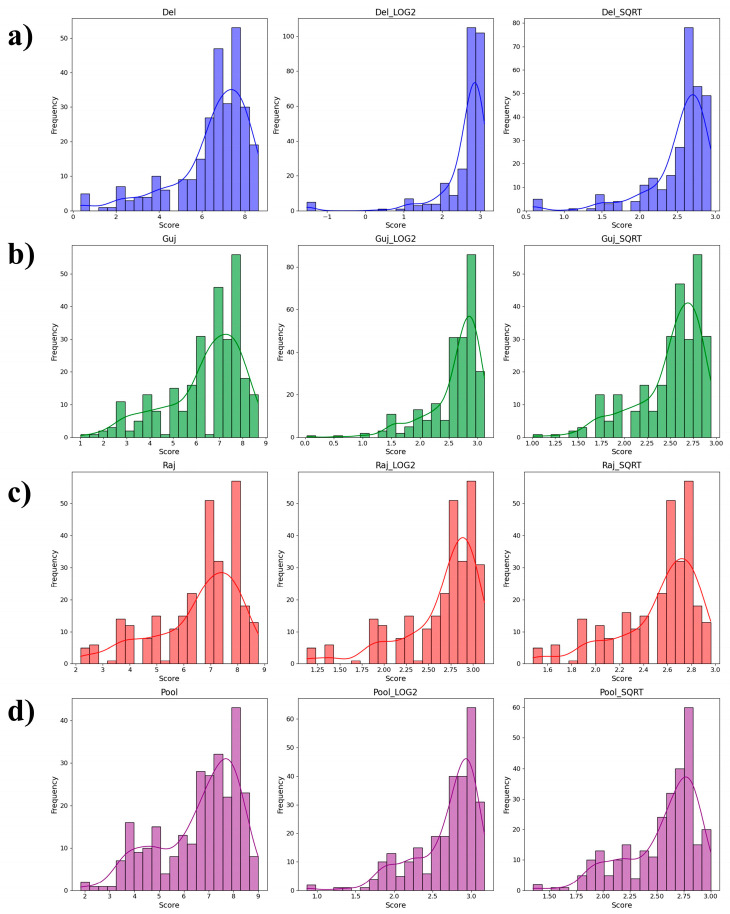
Histograms depicting the frequency distribution of blast scores for (**a**) PMg_Del isolate, (**b**) PMg_Guj isolate, (**c**) PMg_Raj isolate, and (**d**) pooled data. The x-axis represents blast scores, while the y-axis indicates the frequency. log_2_ and SQRT represent logarithmic and square root transformations, respectively.

**Figure 2:**
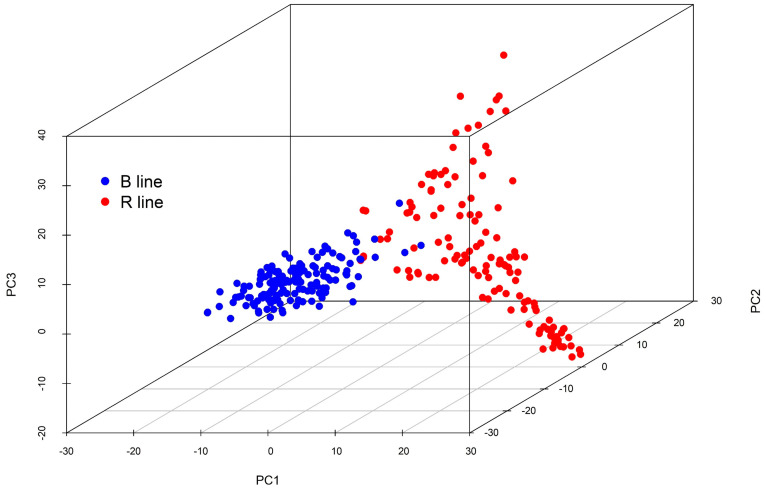
Principal components of 281 inbred lines from genotypic data of 2421 SNPs highlighting the differentiation of B and R lines.

A nearly normal frequency distribution of blast scores was observed for all isolates and pooled data (Figure 1). The mean disease scores were highest for PMg_Del, which exhibited considerable variability in standard deviation among isolates. Additionally, principal component analysis (PCA) revealed distinct clustering of the genotypes into two major groups, each encompassing both B lines and R lines (Figure 2).

### 2.2. Marker-Trait Associations (MTAs)

**Figure 3:**
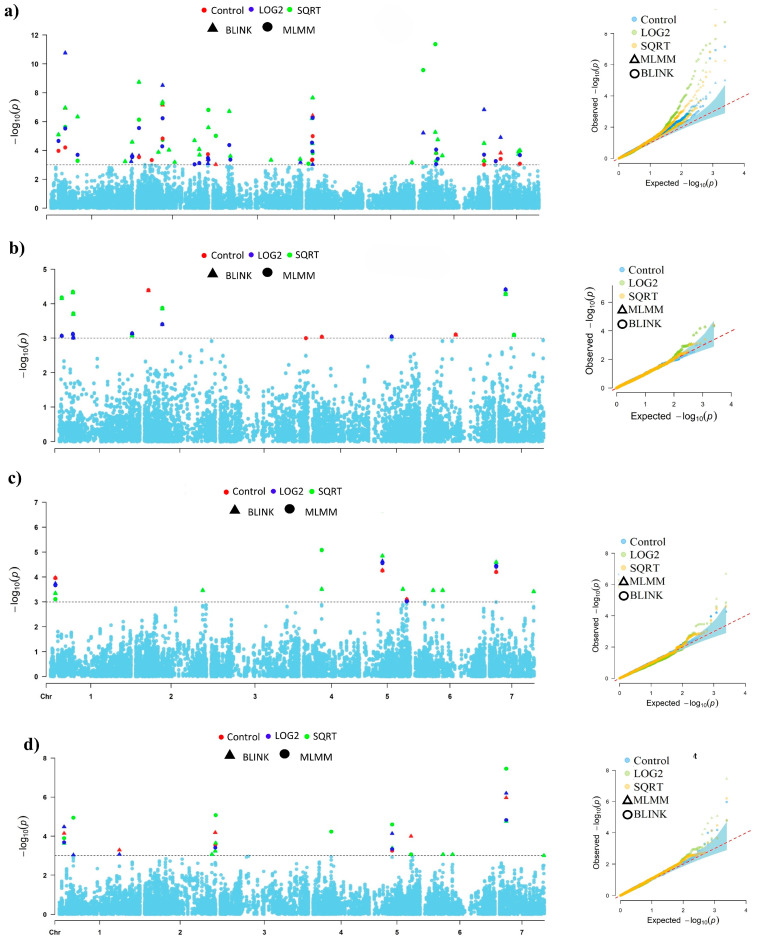
Manhattan plot showing MTAs in (**a**) PMg_Del isolate, (**b**) PMg_Guj isolate, (**c**) PMg_Raj, and (**d**) pool data. The x-axis represents chromosome numbers, while the y-axis displays the −log10(*p*-value) of the association tests. The horizontal dashed line indicates the significance threshold. Different colors represent the data transformations applied: red for control data, blue for log_2−_ transformation, and green for SQRT transformation. Triangle and circle symbols correspond to the statistical models used, with BLINK represented by triangles and MLMM by circles. Their corresponding QQ plots display the expected −log10(*p*-values) versus observed −log10(*p*-values), with deviation from the diagonal line indicating the presence of true associations.

Both Bayesian information and linkage disequilibrium iteratively nested keyway (BLINK) and Multi-Locus Mixed Model (MLMM) models [29] identified 68 SNPs with significant associations (*p* < 0.001) to blast resistance distributed across the 7 chromosomes. The SNP distribution is as follows: chromosome 2 (17 SNPs), chromosome 6 (12 SNPs), chromosome 1 (11 SNPs), chromosome 3 (9 SNPs), chromosome 7 (9 SNPs), chromosomes 4 (5 SNPs), and chromosome 5 (5 SNPs). To enhance the robustness of our findings, SNPs were further filtered using Bonferroni correction, establishing a significance threshold of −log10(*p*-value) > 6.38. Among the identified SNPs, 49 were unique to PMg_Del, 12 were common between PMg_Guj and PMg_Del, and 17 were unique to the pooled data (Table 2). Manhattan plots and quantile–quantile (Q-Q) plots (Figure 3) indicated a deviation of observed *p*-values from expected values for all isolates.

#### 2.2.1. Markers Associated with Blast Resistance in PMg_Del Isolate

Of the 49 significant marker-trait associations (MTAs) identified for the PMg_Del isolate, 20 were identified using the BLINK and 29 by MLMM. MTAs detected by MLMM were spread across all chromosomes. BLINK model identified MTAs on all chromosomes except chromosome 5. BLINK identified 6 SNPs in control data, 14 in log_2−_, and 11 in SQRT transformation, while MLMM detected 16 in control, 39 in log_2_- transformation, and 29 in SQRT transformation. Chromosome 2 had the most MTAs (15 SNPs), while chromosome 5 had the least (1 SNP). Both models identified 12 common MTAs in PMg_Del, including four significant MTAs (*PMSnpB569*, *PMSnp82*, *PMSnp222,* and *PMSnpB1100*) across control, log_2−_, and SQRT transformations with *PMSnp82*, explaining 37.24% phenotypic variance in control. BLINK model identified two MTAs for the log_2-_ and SQRT transformations, and MLMM identified 14.

#### 2.2.2. Markers Associated with Blast Resistance in PMg_Guj Isolate

Twelve MTAs associated with PMg_Guj isolate were identified using both BLINK and MLMM models. These MTAs were distributed across all chromosomes except in chromosomes 3 and 4, with the highest on chromosome 1 (four SNPs), followed by chromosome 7 (three SNPs), chromosome 6 (two SNPs), chromosome 2 (two SNPs), and chromosome 5 (one SNP). Both models consistently identified the same MTAs for blast resistance across control and transformed data sets. Specifically, three MTAs (*PMSnp23*, *PMSnp79*, and *PMSnp82*) were identified in both the log_2−_ and SQRT transformations. Additionally, four MTAs (*PMSnp2330*, *PMSnp586*, *PMSnpB1336*, and *PMSnpB236*) were captured across data sets and GWAS models.

#### 2.2.3. Markers Associated with Blast Resistance in PMg_Raj Isolate

Twelve MTAs associated with the PMg_Raj isolate were identified. BLINK found 11 SNPs, and MLMM detected all 12 MTAs across control, log_2−_, and SQRT transformations. BLINK consistently identified three SNPs (*PMSnp1400*, *PMSnp1871*, and *PMSnpB1692*) in the log_2−_ and SQRT transformations, and three MTAs (*PMSnp2330*, *PMSnpB1336*, and *PMSnpB33*) were significantly associated across control, and log_2−_ and SQRT transformed data. MLMM detected two MTAs (*PMSnp1400* and *PMSnp1826*) in log_2_ and square root transformations as well as five MTAs (*PMSnp1871*, *PMSnp2330*, *PMSnpB1336*, *PMSnpB1692*, and *PMSnpB33*) were significant across all transformations. *PMSnp2330*, *PMSnpB1336*, and *PMSnpB33* were consistently significant across all transformations in both BLINK and MLMM GWAS models, explaining 5.2% (*PMSnpB1336*) to 74.5% (*PMSnp1400*) of phenotypic variance in log_2_-transformed data.

#### 2.2.4. Markers Associated with Blast Resistance in Pooled Data

In the pooled data, seven MTAs were common to both models, while the rest were unique. Chromosome 2 has the highest number of associations (4), followed by chromosomes 5 and 1 (three SNPs each), while chromosomes 3, 6, and 7 had two SNPs each. BLINK identified a highly significant marker, *PMSnp82*, while MLMM captured *PMSnp848* across control, log_2−_, and SQRT transformations. *PMSnp2330*, *PMSnpB33*, and *PMSnpB1336* were consistently identified across control log_2−_ and SQRT transformations and both BLNIK and MLMM models with *PMSnp2330*, explaining 43.60% of phenotypic variance in control. BLINK also identified *PMSnp264* in the control and SQRT transformations and *PMSnpB1484* in the control and log_2-_ transformations. Among the 17 MTAs, 13 were common across the three isolates, with five common to PMg_Del, five common to PMg_Guj, and three common to PMg_Raj. BLINK identified four unique MTAs, namely *PMSnpB1484*, *PMSnp1010*, *PMSnp1007*, and *PMSnp264*, in the pool data.

#### 2.2.5. Significant MTAs Commonly Identified Across Different Isolates

*PMSnp2330* showed strong significance across all three isolates and the pooled data in both GWAS models. *PMSnp82* was common across PMg_Del, PMg_Guj, and the pool, while *PMSnp848* was significant in PMg_Del, PMg_Raj, and pooled data (Table 2). Two MTAs (*PMSnp617* and *PMSnp856*) were detected in PMg_Del and pooled data. *PMSnp2031*, *PMSnp2073*, *PMSnp2575*, *PMSnp819*, and *PMSnpB1336* were strongly associated with blast resistance in PMg_Guj, PMg_Raj, and pooled data. *PMSnp1400*, *PMSnp1826*, and *PMSnpB33* were commonly significant across PMg_Raj and the pooled data. The consistently significant SNPs across both models and transformations indicate robust genetic regions that confer resistance and are useful for accelerated breeding and selection programs.

### 2.3. Identification of Candidate Genes Related to the Blast Resistance in PMg Pathotypes

By leveraging the genome feature file (GFF) of the *P. glaucum* reference, a total of 77 genes located within 1 Mb upstream and downstream region of each significantly associated with blast were identified, highlighting their role in biological processes related to defense mechanisms, including resistance to biotic stress factors, growth regulation, and the biosynthesis of secondary metabolites (Table 3 and Table S1). Notably, several of these genes encode proteins involved in pathogen recognition, signal transduction, and stress response pathways, suggesting their functional relevance in conferring resistance to the blast pathogen.

**Figure 4:**
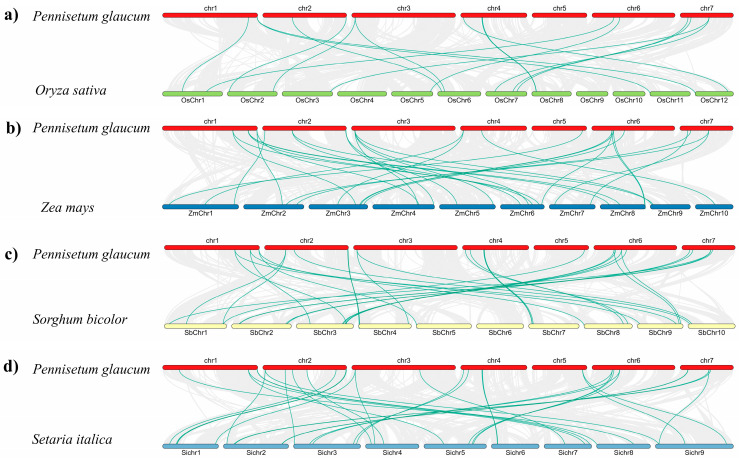
Synteny between *Pennisetum glaucum* blast-resistant genes and their orthologs in (**a**) *Oryza sativa*, (**b**) *Zea mays*, (**c**) *Sorghum bicolor*, and (**d**) *Setaria italica*.

Synteny analysis between blast-resistant genes in *Pennisetum glaucum* (pearl millet) and those in other cereal crops affected by the blast, including *Oryza sativa* (rice), *Zea mays* (maize), *Setaria italica* (foxtail millet), and *Sorghum bicolor* (sorghum), revealed higher sequence similarities (Figure 4). Out of the 77 blast-related genes identified in pearl millet, 21, 23, 35, and 28 showed significant homology with rice, maize, foxtail millet, and sorghum, respectively. *Pgl_GLEAN_10004433* in pearl millet showed 97.72% similarity with multiple regions on chromosome 7 of foxtail millet. *Pgl_GLEAN_10024314* displayed substantial sequence similarity with different regions on rice chromosome 11, with percent identities ranging from 71.6% to 82.7%, indicating strong evolutionary conservation (Table S2). Pgl_GLEAN_10019061 and Pgl_GLEAN_10028688 exhibited more than 80% similarity with genes located on rice chromosomes 1 and 10, respectively. High similarity (91.98%) was observed between *Pgl_GLEAN_10035106* in pearl millet and with rice, emphasizing conserved genetic regions. *Pgl_GLEAN_10000574* shared over 85% similarity with sequences on chromosome 2 in maize.

## 3. Discussion

Foliar blast, caused by *Pyricularia grisea*, is a serious constraint for the successful pearl millet cultivation in arid and semi-arid regions, leading to substantial losses in grain and forage yield. Developing durable resistance is challenging due to the rapid evolution of new pathotypes and the complexity of host–pathogen interactions. The environmental factors often influence the underlying genetic architecture, making it difficult to achieve durable resistance. GWAS offers a powerful approach to identifying loci governing blast resistance, which can be utilized in breeding programs to develop blast-resistant pearl millet cultivars.

A wide range of disease scores among the genotypes highlighted the complex genetic basis of blast resistance. Forty-seven inbreds (16.7%) of the GWAS panel exhibited high resistance, while six were highly susceptible, indicating the presence of valuable genetic resources within the GWAS panel for gene mapping and breeding programs. The analysis of variance revealed significant genotypic effects, indicating that the phenotypic differences were largely due to genetic variation. Additionally, the high heritability estimates affirm the strong genetic control of blast resistance traits and support the potential for effective selection and genetic improvement of resistance traits.

Chromosome 2 showed many MTAs for PMg_Del isolate, while chromosomes 1 and 6 were more important for PMg_Guj and PMg_Raj isolates. Common MTAs, such as *PMSnp2330*, *PMSnp82*, and *PMSnp848*, suggested the presence of broad-spectrum resistance loci that may offer durable resistance across multiple isolates. Identification of isolate-specific markers indicated distinct genetic mechanisms governing resistance to different isolates, highlighting the need to incorporate multiple genes in breeding programs to develop broad-spectrum resistance.

### 3.1. Identification and Functional Categorization of Candidate Genes for Blast Resistance

GWAS analysis identified 68 significant MTAs associated with blast resistance, predominantly located on chromosomes 1, 2, 3, 6, and 7, with fewer loci on chromosomes 4 and 5. Within the identified QTL intervals, 72 candidate genes were identified, which are potentially involved in blast resistance. These genes were associated with various defense mechanisms and stress responses.

#### 3.1.1. Defense and Stress Response Mechanisms Against Pathogens

**Figure 5:**
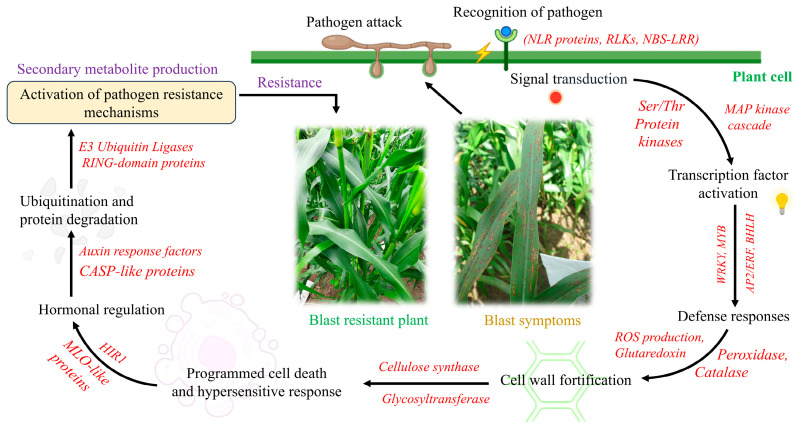
Role of genes and pathways involved in defense mechanisms against blast infection in pearl millet.

Several genes identified were directly involved in defense and stress response mechanisms against pathogens. When the blast fungus infects the crop, the plant recognizes the pathogen through receptors like *NLR proteins* and *cysteine-rich receptor-like protein kinases*. This recognition triggers signal transduction via pathways involving *protein kinases* and *phosphorylation*, leading to the activation of transcription factors such as *WRKY*, *MYB*, *AP2/ERF*, and *BHLH*. These factors regulate the expression of defense-related genes, resulting in *reactive oxygen species* production through *peroxidases* and *catalases*. Additionally, the plant strengthens its cell walls by activating *cellulose synthase* and *glycosyltransferases*. Programmed cell death (PCD) and the hypersensitive response (HR) are initiated to stop the spread of the pathogen, while hormonal regulation through *auxin response factors* fine-tune these processes [30]. Ubiquitination pathways involving *E3 ubiquitin ligases* mark proteins for degradation, ensuring proper protein turnover. Collectively, these processes lead to an enhanced defense response, underscoring the importance of the candidate genes identified in improving resistance to the pathogen (Figure 5).

The *14-3-3-like protein GF14-E*, linked to the SNP, *PMSnp969* is a regulatory protein that modulates stress responses by interacting with proteins such as *BAD* (*Bcl-2-antagonist of cell death*) and *FKHRL-1*, ultimately inhibiting cell death [31]. This protein also activates other defense-related proteins and is involved in both *effector-triggered immunity* (*ETI*) and *pathogen-associated molecular pattern* (*PAMP*)-triggered immunity (PTI). *Hypersensitive-induced response protein 1*, associated with *PMSnpB500*, plays a key role in localized cell death at infection sites and modulates plant resistance through the salicylic acid pathway. In rice, its homologous gene *NbHIR3s* contributes to basal resistance against viral and bacterial pathogens [32]. *EMSY-LIKE protein*, linked to *PMSnp138*, functions as a histone reader that blocks RNA Pol II access to viral genes, suppressing viral gene expression and enhancing basal defense. It is also implicated in *RPP7*-mediated race-specific immunity in Arabidopsis [33]. *MLO-like protein 9*, associated with markers *PMSnp417* and *PMSnpB526*, is known for its role resistance to powdery mildew and may contribute to broader stress responses [34].

Disease-resistance proteins such as *RGA2* (*PMSnp45*), *RGA4*, and RPP8 (*PMSnpB1012*) induce resistance to fungal pathogens by triggering cell death [35]. In rice, *RGA4* is recognized as a blast-resistance gene that triggers cell death upon interaction with the effector *AVR-Pia* of *Magnaporthe oryzae* [36]. Another NLR-encoding gene, ADR1-Like 3, associated with *PMSnpB1066*, is involved in basal immunity and partial resistance to the clubroot pathogen in Arabidopsis [37]. *NDR1/HIN1-like protein 3 (NHL3)*, linked to *PMSnp401*, is a pathogenesis-related gene activating the jasmonic acid signaling pathway, conferring resistance to tobacco mosaic virus in *Nicotiana benthamiana* [38]. The *OsNBL3* gene, encoding a *pentatricopeptide repeat (PPR)* protein and associated with *PMSnp781* and *PMSnp787* on chromosome 2, is essential for mitochondrial development and organelle biogenesis, enhancing resistance to both biotic and abiotic stresses in Arabidopsis [39].

#### 3.1.2. Signal Transduction in Plant Stress Responses

Signal transduction pathways are critical for mediating plant responses to various stress factors. Plants have evolved sophisticated strategies to adapt to biotic stress, primarily through *receptor-like kinases (RLKs)* that detect environmental and pathogenic signals, relaying these signals to regulate growth, development, and stress-responsive mechanisms. *Leucine-rich repeat receptor kinases* (*LRR-RLKs*) are the largest family of *RLKs*, playing dual roles in regulating plant growth and hormone signaling and in mediating defense responses to both biotic and abiotic stresses [40]. Studies in model species such as *Arabidopsis thaliana*, soybean, wheat, and rice have demonstrated that *RLKs*, including those with *LRR domains*, *NBS-LRR-like proteins*, and *LRRNT_2* (*Leucine-rich repeat N-terminal domain*) proteins, play key roles in resistance to viral infections (*PMSnp82*). Wang et al. (2021) identified the blast resistance gene *Pi65* in rice, which contains an LRR domain crucial for conferring resistance against *Magnaporthe oryzae* [41]. Studies in soybeans have shown that disease-resistant genes, including those encoding *LRR-RLKs*, are involved in immune responses, signal transduction, and responses to biotic and abiotic stresses [42].

*FLS2*, a well-characterized *LRR-RLK*, functions as a pattern-recognition receptor (PRR) that binds to *bacterial flagellin* (*flg22*), initiating signal transduction via the *mitogen-activated protein kinase* (*MAPK*) *cascade* and leading to the phosphorylation of intracellular kinases [43]. *EFR*, another cell-surface *RLK* in *Arabidopsis*, recognizes an epitope of bacteria, thereby activating plant defense responses [44]. Protein kinase domain-containing proteins (*PMSnpB1336* and *PMSnp264*) are involved in *jasmonic acid* (JA) and ethylene (ET) signaling, playing an essential role in activating defense mechanisms against stress [45]. Many disease-resistant R genes in plants encode nucleotide-binding site *leucine-rich repeat* (*NBS-LRR*) proteins, which act as receptors within signal transduction pathways activated in response to pathogen attacks. *Pi40(t)* in rice encodes an *NBS-LRR* protein that interacts with pathogen effector genes, following a gene-for-gene model of resistance, conferring resistance to blast [46]. Liu et al. [47] demonstrated that the *NBS-LRR* protein *Pik-h4* interacts with *OsBIHD1* to balance blast resistance and growth by coordinating the ethylene-brassinosteroid pathway in rice.

The *NBS-LRR* family also includes *receptor serine-threonine kinase* (*RSTK*) *proteins*, which are involved in disease resistance, plant development, and self/non-self-discrimination. *RSTK*, a transmembrane protein, regulates these processes alongside other protein families. Receptor-like *protein kinase At4g00960* (*PMSnpB474*) contains a disease resistance-related domain and contributes to stripe rust (*Puccinia striiformis* f. sp. *tritici*) resistance in Sichuan wheat [48]. *Cysteine-rich receptor-like kinases* (*CRKs*) associated with *PMSnp1322* and *PMSnpB1088* are implicated in disease resistance and defense responses in crops such as rice and wheat. In wheat, *TaCRK2* modulates leaf rust resistance, while *ALS1* in rice mediates constitutive defense responses [49,50]. Over-expression of *CRKs* such *as AtCRK5*, *AtCRK6*, *AtCRK13*, *AtCRK28,* and *AtCRK36* enhances pathogen resistance in transgenic *Arabidopsis* [49].

*Serine/threonine protein phosphatases*, including *PP1*, *PP2*, and *BSL2*, associated with markers *PMSnpB236*, *PMSnp640*, *PMSnp586*, *PMSnpB424*, and *PMSnpB1749* regulate triglyceride metabolism and enhance resistance to sheath blight disease in rice [51]. The regulatory protein *TOR 1* (*PMSnp417*) modulates plant defense in response to blast by engaging in crosstalk with multiple signaling pathways [52]. Wall-associated *receptor kinase*, linked to *PMSnp856*, is encoded by the blast-resistant *Pb4* gene and is induced by *chitin and polygalacturonic acid* in rice, contributing positively to defense against infection [53]. *PMSnpB569*, associated with *receptor-like serine/threonine-protein kinase*, an ortholog of *PBL 34*, which plays a role in plant innate immunity [54]. *Phosphatidylinositol 4-kinase gamma 4* (*PI4K*), associated with marker *PMSnp1010*, is an enzyme involved in signaling pathways that regulate the pathogenesis of leaf curl virus in chili, where transient silencing of this gene enhances pathogen resistance [55]. *PI4K* plays a crucial role in *salicylic acid-mediated* plant defense signaling in *Arabidopsis* [56].

*Calcium-dependent protein kinase 2* (*CDPK2*) is critical in calcium signaling for activating defense mechanisms, while *calcineurin B-like protein 2* (*CBL2*) serves as a calcium sensor involved in abiotic stress signaling. *Cysteine-rich receptor-like protein kinase 10* (*CRK10*) is involved in pathogen recognition and signal transduction, highlighting its role in defense responses [57]. *Mitogen-activated protein kinase 5* (*MAPK5*) and *serine/threonine-protein kinases* are involved in downstream signaling pathways regulating stress responses and other cellular processes.

#### 3.1.3. Transcription Factors

*Transcription factors* (*TFs*), such as *AP2/ERF domain-containing proteins*, *BHLH domain-containing proteins*, *homeobox domain-containing proteins*, *MYB*, and *WRKY transcription factor*, play crucial roles in regulating gene expression in response to stress. *AP2/ERF* TFs are known to regulate ethylene-responsive genes, while *BHLH TFs* are involved in various biological processes, including stress responses. For instance, *BHLH TFs* (*PMSnp2402*, *PMSnpB33*, and *PMSnp454*), detected in PMg_Raj isolate and pooled data, activate immune responses against blast infection by regulating *SA/JA signaling* [58]. *Apetala2/ethylene-responsive factor* (AP2/ERF) domain-containing proteins (*PMSnpB629*, *PMSnp1255*, and *PMSnp2469*) regulate plant growth and responses to various stresses and environmental stimuli. *OsERF83* protein positively regulates resistance to *Magnaporthe oryzae* in rice, while the *OsEREBP1* gene activates multiple signaling pathways, including jasmonate and abscisic acid signaling, thereby enhancing survival under biotic and abiotic stress [59,60]. Homeobox domain-containing proteins are key regulators of developmental processes and stress responses.

*PMSnpB1484* and *PMSnpB1692* are associated with *WRKY TF*, a positive regulator of enhanced blast disease resistance in rice by activating the *salicylic acid* (*SA*)-dependent pathway, and its overexpression in plants has been associated with resistance to *Magnaporthe oryzae* [61]. *PMSnpB755* is associated with the *growth-regulating factor* (GRF), a plant-specific transcription factor that may play a role in defense responses. In rice, *miR396* controls *OsGRF* and regulates various biological processes, including floral organogenesis, grain size, yield, and pathogen infection [62]. *miR396* negatively regulates rice immunity against the blast fungus *M. oryzae* by suppressing multiple *OsGRFs*. Blocking *miR396* through target mimicry significantly upregulates *OsGRFs* and leads to enhanced resistance against *M. oryzae*. In sorghum, *SbGRF* responds to pest aphid attacks, indicating its function in the immune response against pests [63].

*BURP domain*-containing plant-specific proteins have diverse roles in plant growth, development, and stress responses. *BURP proteins* (*PMSnp704* and *PMSnp2031*) are involved in stress signal transduction and interact with *MKK* proteins in a conserved manner. In rice, *BURP2* enhances tolerance to drought, salinity, and bacterial blight and increases defense against abiotic stress and bacterial leaf blight [64]. *Homeobox-leucine zipper proteins* (*PMSnp586* and *PMSnp1090*), which are plant-specific TFs, modulate developmental processes and are involved in plant responses to drought, salinity, and pathogen infection. The *ATHB13* gene, which encodes a homeodomain-leucine zipper protein in *Arabidopsis*, and *HDZ27* in pepper positively regulate resistance to fungal pathogens [65]. *PMSnpB1100* is associated with MYB, which contributes to pathogen resistance by regulating defense-related genes. In *Arabidopsis*, *MYB30* enhances the hypersensitive response, a form of programmed cell death that limits pathogen spread [66]. *MYB30* also responds to *microbe-associated molecular patterns* (*MAMPs*) and *ROS* scavenging, leading to the activation of defense pathways that strengthen the plant’s immunity against fungal and bacterial pathogens [67]. By controlling the expression of these defense-related genes. *PMSnp1871* marker associated with *calmodulin-binding transcription activator 3* (*CAMTA3*), known as signal-responsive protein, is involved in mediating biotic stress responses in Arabidopsis [68]. *CAMTA3* plays an important role in defense and plant innate immunity through the modulation of ROS accumulation and SA signaling pathways.

#### 3.1.4. Proteolysis and Protein Modification

Proteolysis and protein modification are essential for maintaining protein quality during stress conditions. The *26S proteasome*, including its non-ATPase regulatory subunit 7 homolog A, is a key component responsible for degrading misfolded or damaged proteins, thereby preserving cellular homeostasis. *Ubiquitin ligases* are critical in tagging these proteins for degradation through the ubiquitin–proteasome pathway, which regulates protein turnover. *Cathepsin B-like protease 2* plays a significant role in protein turnover, further ensuring cellular stability under stress.

*RING-type protein* (*PMSnp79*) interacts with the *AvrPiz-t* effector from *Magnaporthe oryzae*. This interaction promotes the ubiquitination and subsequent degradation of *AvrPiz-t*, thereby positively regulating rice immunity against blast fungus [69]. *RING-type E3 ligases* (*PMSnp617* and *PMSnp1944*) play crucial roles in innate immunity and act as positive regulators of programmed cell death (PCD) and disease resistance in *Arabidopsis* [70]. Genes such as *APIP6/10 and OsBBI1*, which encode *RING finger proteins* with *E3 ligase* activity, mediate broad-spectrum disease resistance in rice [71]. The overexpression of *OsBBI1* in plants has been shown to increase H_2_O_2_ accumulation, enhance phenolic compound levels, and promote the cross-linking of proteins in cell walls at infection sites by *M. oryzae*. This modification of cell wall defense responses underlines *OsBBI1*’s role in broad-spectrum resistance to blast fungus.

*F-box domain-containing proteins*, known for regulating cell cycle, circadian rhythm, development, signal transduction, nutrient sensing, and protein degradation pathways, are significant in this context. F-box protein *OsFBX156* positively regulates defense by mediating the *ubiquitination-dependent degradation of OsHSP71.1* [72]. Other genes such as *MoFwd1*, *MoCdc4*, and *MoFbx15* are known to regulate development and pathogenicity against blast fungus *Magnaporthe oryzae* in rice [73].

#### 3.1.5. Transporters and Oxidation-Reduction Processes

Transporter proteins are essential for the movement of molecules across cellular membranes and play a vital role in stress adaptation. *ABC transporter domain-containing proteins* facilitate the transport of a wide range of substrates, including toxins and signaling molecules [74]. Ammonium transporters are crucial for nitrogen metabolism, while *aquaporin NIP1-1* facilitates the transport of water and small solute transport. Additionally, calcium-transporting *ATPase 4* regulates intracellular calcium levels, which is critical for maintaining cellular homeostasis during stress [75].

*Alcohol dehydrogenase-like 5* participates in oxidation-reduction reactions, while *L-ascorbate oxidase* is involved in ascorbate metabolism and the regulation of reactive oxygen species, underscoring the importance of redox homeostasis in blast resistance [76]. *ROS modulator 1* (*PMSnpB474*) is crucial for controlling *ROS* levels during pathogen attacks. The *1-Cys peroxiredoxin PER1* acts as an antioxidant enzyme and protects cells from oxidative damage by reducing peroxides [77].

*PMSnp848*, associated with catalase, scavenges H_2_O_2_ and is involved in cell wall resistance in plants, serving as a signal for the induction of defense-related genes. *PMSnp819*, associated with peroxidase, is classified as pathogen-related genes [78]. It is activated in response to biotic and abiotic stress and is essential for suppressing blast disease incidence in finger millet [79]. *L-ascorbate peroxidase* (*PMSnp1826*), a ROS scavenger, contributes to H_2_O_2_ removal and mediates host defense mechanisms against fungal infection [80]. With regard to *Glutaredoxin-6* (*PMSnp2330* and *PMSnp949*), *small oxidoreductases* involved in redox regulation and oxidative stress resistance interact with various signaling molecules, including PR genes, providing broad-spectrum resistance to *Xanthomonas oryzae* pv. *oryzae* and *Fusarium fujikuroi* in rice [81].

#### 3.1.6. Metabolism

Metabolic processes are critical for plant growth and defense mechanisms. *PMSnp401*, associated with the gene *Far-red impaired response 1* (*FAR1*), enhances plant immunity in *Arabidopsis* by binding to the promoter region of *HEMB1* and activating its expression, thereby regulating chlorophyll biosynthesis and salicylic acid (SA) signaling pathways [82]. *Glutathione S-transferase* (*PMSnp2014*) detoxifies xenobiotics and protects cells from oxidative damage. This enzyme plays a vital role in regulating the expression of protective genes in infected cells, thereby contributing to plant disease resistance [83]. *PMSnp848*, linked to the gene *Phospholipase D* (*PLD*), is involved in cellular and biological processes in response to biotic and abiotic stimuli, as well as plant–microbe interaction. In pathogen-infected plants, PLD accumulates to regulate defense response by controlling actin cytoskeleton dynamics [84]. *1-aminocyclopropane-1-carboxylate oxidase 1* is essential for ethylene biosynthesis, which modulates stress responses [85]. *Glutathione reductase* maintains the redox state of glutathione, an important antioxidant, while malate dehydrogenase catalyzes the reversible oxidation of malate to oxaloacetate, a critical step in the citric acid cycle that supports energy production and metabolic balance [86].

#### 3.1.7. Structural Proteins and Molecular Chaperones

Structural proteins are essential for maintaining cell integrity and function. *Actin-related protein 3* is part of the cytoskeleton, crucial for cell structure and movement. *Alpha-amylase type A isozyme* serves as an enzyme that breaks down starches, highlighting its role in carbohydrate metabolism and energy provision during stress responses. *Kinesin-like proteins* (*PMSnp1894*) contribute to tolerance against viral and fungal infections in plants. Overexpression of *kinesin-like protein* with *loose plant architecture 1* (*LPA-1*) activates *PINa-dependent auxin* redistribution, leading to subsequent activation of auxin signaling pathways that enhance resistance to sheath blight disease in rice [87]. *PMSnp2575* is associated with *Jumonji C*, a histone demethylase that plays a crucial role in reducing fungal virulence and regulates stress response in rice [88].

Molecular chaperones, including *heat shock protein 90-6* and *chaperone protein dnaJ C76*, play a crucial role in assisting protein folding and protection under stress conditions. These chaperones ensure proper protein conformation and prevent aggregation, which is critical for maintaining cellular function during stress. *BAG family molecular chaperone regulators 7 and 1*, associated with MTAs *PMSnp856* and *PMSnp965*, trigger programmed cell death under infection and enhance disease resistance by activating pathogen-associated molecular pattern-triggered immunity [89].

#### 3.1.8. Secondary Metabolism and Hormone Signaling

*Anthranilate O-methyltransferase 2* and caffeic acid 3-O-methyltransferase, linked to *PMSnpB588* and *PMSnp1400*, respectively, play significant roles in the biosynthesis of secondary metabolites, including lignin and phenylpropanoids [90]. These compounds are essential for strengthening the cell wall and providing defense against pathogens. *Flavonoid O-methyltransferase* participates in flavonoid biosynthesis, further contributing to the plant’s chemical defense arsenal [91].

Hormone signaling pathways regulate various aspects of plant growth and stress responses. *PMSnpB1733*, *PMSnp2021*, *PMSnpB1995*, and *PMSnp2023* are associated with the *auxin response factor* modulates gene expression in response to auxin, a hormone that influences growth and development, playing roles in adjusting plant physiology to cope with stress [92]. *Glycosyltransferases* (*PMSnp2356* and *PMSnp2469*) are crucial for plant tolerance to various stresses, synthesizing secondary metabolites, and regulating plant hormone balance. The maize *glycosyltransferase UFGT2* modifies kaempferol and quercetin, enhancing plant tolerance to abiotic stress [93]. Recent studies identified that *UDP-glycosyltransferase OsUGT706E2* in rice and *UGT87E7* in *Camellia sinensis* regulate tolerance to biotic stress [94,95]. *UGT71C3* regulates SA levels in plants by glycosylating *MeSA*, thereby modulating the plant systemic acquired tolerance and stress responses [96]. *UGT74F* and *UGT74F2* can glycosylate *A*, contributing to plant disease resistance responses.

#### 3.1.9. Cell Wall Modification

Cell wall integrity is crucial for plant defense against pathogens. *Cellulose synthase A catalytic subunit* (*PMSnpB2031*) plays a central role in cellulose synthesis, a major component of the cell wall that provides structural support and resistance to pathogen invasion [97]. Cell wall modification, including expansion, facilitates cell growth and enhances the physical barrier against pathogens. Key genes such as *beta-galactosidase 5***,** which is involved in carbohydrate metabolism, and *lipid phosphate phosphatase 3*, which contributes to lipid metabolism and signaling, are significant in cell wall modification [98,99].

*Patatin* (*PMSnp339* and *PMSnp1964*) is implicated in maintaining cell wall integrity by regulating fatty acid metabolism critical for *jasmonic acid* (*JA*) biosynthesis and JA signaling pathways, thus enhancing pathogen resistance [100]. *CASP-like proteins* (*PMSnp2073* and *PMSnp23*) act as diffusion barriers and are involved in cell wall modification by interacting with peroxidases to mediate lignin deposition [101]. *Lignin* acts as a physical barrier that restricts pathogen movement and contributes to plant resistance. *PMSnpB588*, associated with *xyloglucan endotransglucosylase/hydrolase*, is a key enzyme that regulates cell wall structure and helps plants adapt to stress [102]. *Trichome birefringence-like 24* (*PMSnp1007*) in *Arabidopsis* regulates cellulose and pectin deposition in the cell wall, thereby providing resistance to powdery mildew by limiting fungal growth [103].

Comparative analysis of the candidate genes identified in pearl millet offers insights into the evolutionary relationship of blast resistance mechanisms in other cereal crops. High similarity (70–97%) in the blast-related genes between pearl millet with rice, maize sorghum, and foxtail millet suggests that these genes have been conserved through evolution, likely because they play crucial roles in the plants’ defense mechanisms against fungal pathogens [104]. These findings suggest that the conserved genes identified in pearl millet may play crucial roles in blast resistance across cereal crops, providing valuable targets for functional genomics and breeding programs aimed at improving blast resistance.

## 4. Materials and Methods

### 4.1. GWAS Panel

This study utilized a diverse panel of 281 pearl millet inbred lines sourced from the pearl millet breeding program at ICAR-Indian Institute of Millets Research, Hyderabad (Table S3). The panel comprised 154 B lines and 127 R lines, comprehensively representing genetic variability for the investigation.

### 4.2. Genomic DNA Extraction

Genomic DNA was isolated from 10 to 12 seeds from each of the 281 pearl millet inbreds of the GWAS panel, using the CTAB (Cetyl Trimethyl Ammonium Bromide) extraction method [105]. The quantity and quality of the isolated DNA were assessed using a spectrophotometer and Nanodrop 1000 (Thermo Scientific, Waltham, MA, USA), measuring absorbance at 260 and 280 nm. Qualitative analysis was performed using agarose gel electrophoresis (1% agarose gel, 80 V for 30 min), and the bands were visualized with a gel documentation system to check for RNA and protein contamination.

### 4.3. Phenotyping

Pathogen inoculation was performed using three *Pyricularia grisea* isolates: PMg_Del (Delhi), PMg_Raj (Rajasthan), and PMg_Guj (Gujarat). These isolates were selected for their relevance to regional disease dynamics. The inoculation was conducted on 15-day-old seedlings of the GWAS panel grown in cups, which were at the three-leaf stage. An aqueous conidial suspension of *P. grisea* was prepared at a concentration of approximately 1 × 10^5^ spores mL^−1^. The suspension was uniformly sprayed onto the foliage using a handheld sprayer to ensure complete coverage until runoff, maximizing spore contact with the leaf surfaces. After inoculation, the plants were monitored, and disease incidence was evaluated ten days post-inoculation using a 0–9 scale [13,106]: 0–1 (highly resistant), 2–3 (moderately resistant), 4–5 (moderately susceptible), 6–7 (susceptible), and 8–9 (highly susceptible).

### 4.4. Genotyping

An AgriSeq 4K mid-density SNP panel [19], comprising 4072 SNPs, was used to generate genotypic data through the targeted-genotype by sequencing (tGBS) approach from 281 pearl millet inbreds [19]. After removing monomorphic SNPs and applying filters such as heterozygosity (>10%), missing values (>10%), and minor allele frequency (<2%), a total of 2421 high-confidence SNPs distributed across seven chromosomes were retained. This high-quality SNP dataset was used for subsequent association mapping analysis.

### 4.5. Phenotypic Data Analysis

Descriptive statistics such as maximum, minimum, range, and mean values were calculated for all three isolates. The adjusted means of replicates for each isolate were obtained by fitting mixed linear models (MLM) for each genotype. These adjusted means, calculated as best linear unbiased predictions (BLUPs), were derived by considering replication and genotype as random effects. BLUPs for each genotype, along with the analysis of variance (ANOVA), were estimated using the R package *lme4* v.1.1 [107]. The BLUPs were calculated using the following formula [108].

$$Y_{ik}=\mu +R_{i\;}+G_{k}+\epsilon_{ik}$$

where *Y_ik_* is the trait of interest; *μ* is the mean effect; *R_i_* is the effect of the ith replicate; *G_k_* is the effect of the kth genotype; *ϵ_ik_* is the error associated with the ith replication and the kth genotype, which is assumed to be normally and independently distributed, with mean zero and homoscedastic variance *σ*^2^. The broad sense heritability (*h*^2^) was estimated using the formula [109].

$$h^{2}=\frac{\sigma_{g}^{2}}{\sigma_{g}^{2}+\sigma_{e}^{2}\;/nreps}$$

where $\sigma_{g}^{2}$ is the genotypic variance; $\sigma_{e}^{2}$ is the error variance, and *nreps* is the number of replications.

### 4.6. Genome-Wide Association Mapping (GWAS)

For the GWAS analysis, 2421 filtered SNPs and the BLUPs derived from the data of the three isolates and the pooled mean values were used. Considering that the data are semi-quantitative, it is essential to normalize the trait distribution to satisfy the underlying assumptions of GWAS models [110]. Hence, we implemented data transformations, namely log_2_ and square root (SQRT) transformations, alongside a control (the non-transformed original BLUP values) data set [110]. These transformations were employed to normalize the data and stabilize variance, thereby improving the accuracy and reliability of the GWAS results. Two models, namely MLMM (Multi-Locus Mixed Model) and BLINK (Bayesian information and linkage disequilibrium iteratively nested keyway) were employed for identifying marker-trait associations (MTAs) using Genome Association and Prediction Integrated Tool (GAPIT) version 3.1.0 in the R [29]. The complementary use of MLMM and BLINK models balances the control of false positives with the detection of true associations, making them well-suited to the complex genetic landscape of blast resistance in pearl millet. Principal component analysis (PCA) and kinship relationships among genotypes were calculated using GAPIT to account for population structure and were integrated into the GWAS models to adjust for population stratification [111].

### 4.7. Identification of Candidate Genes

Significant SNPs identified through GWAS were mapped to their corresponding positions on the reference genome [112]. The genomic regions spanning 1 Mb upstream and downstream of each significantly associated SNP were explored to identify potential candidate genes for foliar blast.

### 4.8. In Silico Comparative Analysis

Significant SNPs identified through the BLINK and MLMM models were searched for orthologous genes in other monocot species. These species include rice (*Oryza sativa*), maize (*Zea mays*), sorghum (*Sorghum bicolor*), and foxtail millet (*Setaria italica*), all of which are affected by foliar blast. Local alignments were performed against the genomes of these species to identify orthologous sequences corresponding to the significantly associated SNPs. The alignments were subjected to a stringent threshold (*E* ≤ 0.01) to ensure high confidence. TBtools was used for sequence alignment, employing the MCScanX toolkit to visualize synteny and ortholog relationships [113,114].

## 5. Conclusions

This study successfully identified significant phenotypic variation for blast resistance among the 281 pearl millet inbreds, with clear distinctions between highly resistant and highly susceptible genotypes. GWAS revealed 68 significant MTAs, highlighting key regions involved in blast resistance. SNPs such as *PMSnp82* and *PMSnp2330* showed consistent associations across multiple isolates and in the pooled data, indicating their importance in blast resistance. The identification of 77 candidate genes associated with significant MTAs provided valuable insights into the biological pathways associated with disease resistance. Many of these genes are involved in pathogen recognition, signal transduction, and stress response mechanisms, further emphasizing their functional relevance in conferring blast resistance.

Identified defense-related genes, including those involved in *NLR recognition*, *WRKY* and *MYB transcription factors*, and reactive oxygen species production, highlight the complex nature of blast resistance in pearl millet. Synteny analysis between *Pennisetum glaucum* and other cereal crops such as rice, maize, sorghum, and foxtail millet revealed conserved genomic regions. These findings suggest that several genes in pearl millet may play crucial roles in blast resistance across multiple cereal crops, providing promising targets for breeding programs aimed at improving disease resistance. Our research revealed key genetic markers and candidate genes that can be leveraged in breeding and gene-editing programs for enhancing the resilience of pearl millet and other cereal crops to blast disease, thereby contributing to improved crop productivity and food security.

## Figures and Tables

**Table 1:** Analysis of variance and heritability for blast resistance across different isolates of pearl millet.

Isolates	Source	Df	Sum Sq	Mean Sq	F Value	Heritability (%)
PMg_Del	Genotype	280	1195.410	4.269	6.349 **	86.3
	Replication	1	0.001	0.001	0.002	
	Residual	261	175.498	0.672		
PMg_Guj	Genotype	280	1663.520	5.941	9.489 **	90.5
	Replication	1	1.320	1.320	2.109	
	Residual	275	172.179	0.626		
PMg_Raj	Genotype	280	1622.990	5.796	9.697 **	90.7
	Replication	1	1.835	1.835	3.070	
	Residual	278	166.164	0.597		
Pool	Genotype	280	3778.158	13.493	15.015 **	93.8
	Replication	1	2.771	2.771	3.084	
	Residual	1373	1233.828	0.898		

** significance at *p* = 0.01.

**Table 2:** Number of MTAs co-mapped across different isolates of *Pyricularia grisea* in pearl millet using BLINK and MLMM models.

Sl. No.	Traits	No. of MTAs (BLINK)	No. of MTAs (MLMM)	Common MTAs
1	PMg_Del_Control	8	16	6
2	PMg_Del_log_2_	14	39	9
3	PMg_Del_SQRT	11	29	7
4	PMg_Guj_Control	4	4	4
5	PMg_Guj_log_2_	12	12	12
6	PMg_Guj_SQRT	7	7	7
7	PMg_Raj_Control	6	6	6
8	PMg_Raj_log_2_	4	11	4
9	PMg_Raj_SQRT	10	7	7
10	PMg_Pool_Control	7	4	4
11	PMg_Pool_log_2_	9	13	13
12	PMg_Pool_SQRT	5	4	4

**Table 3:** Common SNPs identified through BLINK and MLMM models across isolates with their associated genes and functions.

Sl. No.	SNP Name	Isolate Name	Linked Gene(s)	Gene Annotation	Resistance Mechanism
1	*PMSnp82*	PMg_Del_Control PMg_Del_log_2_ PMg_Del_SQRT	*Pgl_GLEAN_10023779*	*LRRNT_2 domain-containing protein*	Key roles in resistance to pathogen attack
2	*PMSnpB1100*	PMg_Del_Control PMg_Del_log_2_ PMg_Del_SQRT	*Pgl_GLEAN_10032560*	*Putative cysteine-rich receptor-like protein kinase 35*	Involved in pathogen recognition and signal transduction
			*Pgl_GLEAN_10025439*	*Transcription factor MYB83*	Play crucial role in regulating gene expression in response to stress
3	*PMSnp2222*	PMg_Del_Control PMg_Del_log_2_ PMg_Del_SQRT	*Pgl_GLEAN_10019061*	*Probable LRR receptor-like kinase At3g47570*	Hormone signaling and mediating defense responses
4	*PMSnpB236*	PMg_Guj_Control PMg_Guj_log_2_ PMg_Guj_SQRT	*Pgl_GLEAN_10025523*	*Serine/threonine-protein phosphatase PP1*	Involved in signaling pathways regulating stress responses and cellular processes
5	*PMSnp586*	PMg_Guj_Control PMg_Guj_log_2_ PMg_Guj_SQRT	*Pgl_GLEAN_10022625*	*Homeobox-leucine zipper protein*	Modulate developmental processes and are involved in plant responses to drought, salinity, and pathogen infection
			*Pgl_GLEAN_10022624*	*Probable serine/threonine-protein kinase PBL3*	Role in plant innate immunity
6	*PMSnpB33*	PMg_Raj_Control PMg_Raj_log_2_ PMg_Raj_SQRT PMg_Pool_Control PMg_Pool_log_2_ PMg_Pool_SQRT	*Pgl_GLEAN_10017646*	*BHLH domain-containing protein*	Role in regulating gene expression in response to stress
7	*PMSnpB1336*	PMg_Raj_Control PMg_Raj_log_2_ PMg_Raj_SQRT PMg_Pool_Control PMg_Pool_log_2_ PMg_Pool_SQRT	*Pgl_GLEAN_10005235*	*Protein kinase domain-containing protein*	Involved in jasmonic acid and ethylene signaling
8	*PMSnp2330*	PMg_Raj_Control PMg_Raj_log_2_ PMg_Raj_SQRT PMg_Pool_Control PMg_Pool_log_2_ PMg_Pool_SQRT	*Pgl_GLEAN_10031009*	*Receptor-like serine/threonine-protein*	Involved in signaling pathways regulating stress responses and cellular processes
			*Pgl_GLEAN_10008778*	*Glutaredoxin domain-containing protein*	Involved in redox regulation and oxidative stress resistance, interact with various signaling molecules, including PR genes, providing broad-spectrum resistance

## Data Availability

The original contributions presented in the study are included in the article and Supplementary Materials; further inquiries can be directed to the corresponding authors.

## Supplementary Materials

The following supporting information can be downloaded at: https://www.mdpi.com/article/10.3390/ijms252212048/s1.
