# Supplementary material for: Novel SNPs Linked to Blast Resistance Genes Identified in Pearl Millet Through Genome-Wide Association Models
Source: Int J Mol Sci. 2024 Nov 9;25(22):12048. doi: 10.3390/ijms252212048 (PMC11593765; doi:10.3390/ijms252212048)
Supplement: Supplementary file 1 [file ijms-25-12048-s001.zip › ijms-3289181-supplementary.pdf]

Table S1: Functional characterization of candidate genes associated with significant markers for blast resistance

Sl. No	SNP	Allele	Chr	Pos	Gene ID	Gene Annotation	Gene ontology (GO)
1	*PMSnp23*	A/G	1	8062896	*Pgl_GLEAN_10017120*	*CASP-like protein*	Integral component of membrane [GO:0016021]; plasma membrane [GO:0005886]; integral component of membrane [GO:0016021]; plasma membrane [GO:0005886]circadian rhythm [GO:0007623]; hydrogen peroxide catabolic process [GO:0042744]; response to abscisic acid [GO:0009737]; response to bacterium [GO:0009617]; response to heat [GO:0009408]; response to hydrogen peroxide [GO:0042542]; response to light stimulus [GO:0009416]; response to salt [GO:1902074]; response to water deprivation [GO:0009414]; catalase activity [GO:0004096]; heme binding [GO:0020037]; metal ion binding [GO:0046872]
2	*PMSnpB33*	C/A	1	16205605	*Pgl_GLEAN_10017646*	*BHLH domain-containing protein*	Nucleus [GO:0005634]; DNA-binding transcription activator activity, RNA polymerase II-specific [GO:0001228]; protein dimerization activity [GO:0046983] nucleus [GO:0005634]cell surface receptor signaling pathway [GO:0007166]; protein phosphorylation [GO:0006468]ATP binding [GO:0005524]; calcium ion binding [GO:0005509]; polysaccharide binding [GO:0030247]; protein serine/threonine kinase activity [GO:0004674]
3	*PMSnp45*	T/C	1	24642914	*Pgl_GLEAN_10028542*	*Disease resistance protein RGA2*	ADP binding [GO:0043531]defense response to fungus [GO:0050832] ATP binding [GO:0005524]; protein serine/threonine kinase activity [GO:0004674]
4	*PMSnp79*	C/A	1	46462235	*Pgl_GLEAN_10003226*	*RING-type domain-containing protein*	Metal ion binding [GO:0046872]; ubiquitin-protein transferase activity [GO:0004842]metal ion binding [GO:0046872]; ubiquitin-protein transferase activity [GO:0004842]
5	*PMSnp82*	G/A	1	47810451	*Pgl_GLEAN_10023779*	*LRRNT_2 domain-containing protein*	Integral component of membrane [GO:0016021]integral component of membrane [GO:0016021]defense response [GO:0006952]ADP binding [GO:0043531]; ATP binding [GO:0005524]
6	*PMSnp138*	G/A	1	89822439	*Pgl_GLEAN_10004062*	*protein EMSY-LIKE 3*	nucleusdefense response to fungus
7	*PMSnp264*	G/T	1	204844042	*Pgl_GLEAN_10011010*	*Protein kinase domain-containing*	Integral component of membrane [GO:0016021]; ATP binding [GO:0005524]; protein kinase activity [GO:0004672]integral component of membrane [GO:0016021]cell wall biogenesis [GO:0042546]; cell wall organization [GO:0071555]; xyloglucan metabolic process [GO:0010411]hydrolase activity, hydrolyzing O-glycosyl compounds [GO:0004553]; xyloglucan:xyloglucosyl transferase activity [GO:0016762]

8	*PMSnpB236*	A/G	1	248811621	*Pgl_GLEAN_10025523*	*serine/threonine-protein phosphatase PP1*	Nucleus [GO:0005634]; cytoplasm [GO:0005737]protein serine/threonine phosphatase activity [GO:0004722]; hydrolase activity [GO:0016787]
9	*PMSnp339*	G/A	1	250968972	*Pgl_GLEAN_10022383*	*Patatin*	Hydrolase activity [GO:0016787]; lipid catabolic process [GO:0016042] defense response [GO:0006952]
10	*PMSnp401*	T/A	1	271921404	*Pgl_GLEAN_10038305*	*Protein FAR1-RELATED SEQUENCE*	Nucleus [GO:0005634]; zinc ion binding [GO:0008270]; regulation of transcription, DNA-templated [GO:0006355]nucleus [GO:0005634]; plasmodesma [GO:0009506]regulation of DNA-templated transcription [GO:0006355] protein binding [GO:0005515]; metal ion binding [GO:0046872]
11	*PMSnp417*	G/T	1	274864238	*Pgl_GLEAN_10004433*	*regulatory-associated protein of TOR 1*	TORC1 complex [GO:0031931]; TOR signaling [GO:0031929]TORC1 complex [GO:0031931]embryo development ending in seed dormancy [GO:0009793];chromatin remodeling [GO:0006338]; TOR signaling [GO:0031929]
12	*PMSnp454*	C/T	2	6994157	*Pgl_GLEAN_10033865*	*BHLH domain-containing protein*	Nucleus [GO:0005634]; protein dimerization activity [GO:0046983]; internode patterning [GO:0080006]; negative regulation of red or far-red light signaling pathway [GO:0090229]; positive regulation of transcription, DNA-templated [GO:0045893]nucleus [GO:0005634]auxin-activated signaling pathway [GO:0009734]; regulation of transcription, DNA-templated [GO:0006355]DNA binding [GO:0003677]
13	*PMSnpB424*	C/T	2	27380833	*Pgl_GLEAN_10002097*	*serine/threonine-protein phosphatase BSL2 homolog*	Nucleus [GO:0005634]brassinosteroid mediated signaling pathway [GO:0009742]myosin phosphatase activity [GO:0017018]; metal ion binding [GO:0046872]
14	*PMSnpB474*	G/C	2	44966203	*Pgl_GLEAN_10028688*	*putative receptor-like protein kinase At4g00960*	glutathione metabolic process [GO:0006749]glutathione transferase activity [GO:0004364]
15	*PMSnpB500*	G/C	2	50447841	*Pgl_GLEAN_10032282*	*hypersensitive-induced response protein 1*	Internode patterning [GO:0080006]; negative regulation of red or far-red light signaling pathway [GO:0090229]; positive regulation of transcription, DNA-templated [GO:0045893]protein dimerization activity [GO:0046983]
16	*PMSnp586*	C/G	2	60879605	*Pgl_GLEAN_10022625*	*Homeobox-leucine zipper protein*	Nucleus [GO:0005634]; DNA-binding transcription factor activity, RNA polymerase II-specific [GO:0000981]; sequence-specific DNA binding [GO:0043565]nucleus [GO:0005634]methylation [GO:0032259]caffeate O-methyltransferase activity [GO:0047763]; protein dimerization activity [GO:0046983]
17	*PMSnp586*	C/G	2	60879605	*Pgl_GLEAN_10022624*	*probable serine/threonine-protein kinase PBL3*	Nucleus [GO:0005634];cytoplasm [GO:0005737]; plasma membrane [GO:0005886]defense response [GO:0006952]; chromatin remodeling [GO:0006338] protein kinase activity [GO:0004672]; protein serine/threonine kinase activity[GO:0004674]

18	*PMSnpB526*	G/T	2	64507134	*Pgl_GLEAN_10001581*	*MLO-like protein*	Integral component of membrane [GO:0016021]; calmodulin binding [GO:0005516]; defense response [GO:0006952]; response to biotic stimulus [GO:0009607]integral component of membrane [GO:0016021]defense response [GO:0006952]; response to biotic stimulus [GO:0009607]calmodulin binding [GO:0005516]
19	*PMSnp617*	C/T	2	73160230	*Pgl_GLEAN_10021152*	*probable E3 ubiquitin-protein ligase XBOS36*	Transferase activity [GO:0016740]DNA-binding transcription factor activity, RNA polymerase II-specific [GO:0000981]; sequence-specific DNA binding [GO:0043565]
20	*PMSnp640*	C/T	2	86015103	*Pgl_GLEAN_10021949*	*serine/threonine-protein phosphatase PP1*	Nucleus [GO:0005634]; cytoplasm [GO:0005737]protein serine/threonine phosphatase activity [GO:0004722]; hydrolase activity [GO:0016787]
21	*PMSnpB569*	T/A	2	87275137	*Pgl_GLEAN_10028979*	*receptor-like serine/threonine-protein kinase At3g01300*	ATP binding [GO:0005524]; protein serine/threonine kinase activity [GO:0004674]
22	*PMSnpB588*	A/C	2	109063420	*Pgl_GLEAN_10015493*	*Xyloglucan endotransglucosylase/hydrolase*	Apoplast [GO:0048046]; cell wall [GO:0005618]; hydrolase activity, hydrolyzing O-glycosyl compounds [GO:0004553]; xyloglucan:xyloglucosyl transferase activity [GO:0016762]; cell wall biogenesis [GO:0042546]; cell wall organization [GO:0071555]; xyloglucan metabolic process [GO:0010411]apoplast [GO:0048046]; cell wall [GO:0005618]DNA-binding transcription activator activity, RNA polymerase II-specific [GO:0001228]; protein dimerization activity [GO:0046983]
23	*PMSnpB588*	A/C	2	109063420	*Pgl_GLEAN_10015507*	*anthranilate O-methyltransferase 2*	Involved in methylation [GO:0032259]; enables S-adenosylmethionine-dependent methyltransferase activity [GO:0008168]
24	*PMSnp704*	T/A	2	128368921	*Pgl_GLEAN_10036899*	*BURP domain-containing protein*	Regulation of transcription, DNA-templated [GO:0006355]; zinc ion binding [GO:0008270]
25	*PMSnpB629*	C/T	2	195717587	*Pgl_GLEAN_10030508*	*AP2/ERF domain-containing protein*	Nucleus [GO:0005634]; DNA binding [GO:0003677]; DNA-binding transcription factor activity [GO:0003700]; nucleus [GO:0005634]; recognition of pollen [GO:0048544]; ATP binding [GO:0005524]; protein serine kinase activity [GO:0106310]; protein threonine kinase activity [GO:0106311]
26	*PMSnp781*	C/T	2	211741286	*Pgl_GLEAN_10000574*	*pentatricopeptide repeat-containing protein At4g14850*	Intracellular membrane-bounded organelle [GO:0043231]; mitochondrion [GO:0005739]; RNA binding [GO:0003723]; zinc ion binding [GO:0008270]; RNA modification [GO:0009451]; intracellular membrane-bounded organelle [GO:0043231]; mitochondrion [GO:0005739]; RNA modification [GO:0009451;]; RNA binding [GO:0003723]; zinc ion binding [GO:0008270]
27	*PMSnp787*	T/C	2	212722669	*Pgl_GLEAN_10000574*	*pentatricopeptide repeat-containing protein At4g14850*	Intracellular membrane-bounded organelle [GO:0043231]; mitochondrion [GO:0005739]; RNA binding [GO:0003723]; zinc ion binding [GO:0008270]; RNA modification [GO:0009451]intracellular membrane-bounded organelle

							[GO:0043231]; mitochondrion [GO:0005739]; RNA modification [GO:0009451]; RNA binding [GO:0003723]; zinc ion binding [GO:0008270]
28	*PMSnp819*	T/C	2	229749286	*Pgl_GLEAN_10024704*	*Peroxidase*	Extracellular region [GO:0005576]; plant-type cell wall [GO:0009505]; plasmodesma [GO:0009506]; heme binding [GO:0020037]; metal ion binding [GO:0046872]; peroxidase activity [GO:0004601]; hydrogen peroxide catabolic process [GO:0042744]; response to oxidative stress [GO:0006979]extracellular region [GO:0005576]; plant-type cell wall [GO:0009505]; plasmodesma [GO:0009506]
29	*PMSnp848*	A/G	2	241012478	*Pgl_GLEAN_10018073*	*Catalase*	Cytoplasm [GO:0005737]; peroxisome [GO:0005777]; plasma membrane [GO:0005886]; catalase activity [GO:0004096]; heme binding [GO:0020037]; metal ion binding [GO:0046872]; circadian rhythm [GO:0007623]; hydrogen peroxide catabolic process [GO:0042744]; response to abscisic acid [GO:0009737]; response to bacterium [GO:0009617]; response to heat [GO:0009408]; response to hydrogen peroxide [GO:0042542]; response to light stimulus [GO:0009416]; response to salt [GO:1902074]; response to water deprivation [GO:0009414]cytoplasm [GO:0005737]; peroxisome [GO:0005777]; plasma membrane [GO:0005886]lipid catabolic process [GO:0016042]N-acylphosphatidylethanolamine-specific phospholipase D activity [GO:0070290]; phospholipase D activity [GO:0004630]
30	*PMSnp848*	A/G	2	241012478	*Pgl_GLEAN_10018178*	*Phospholipase D*	N-acylphosphatidylethanolamine-specific phospholipase D activity [GO:0070290]; phospholipase D activity [GO:0004630]; lipid catabolic process [GO:0016042]transferase activity [GO:0016740]
31	*PMSnp856*	T/C	2	242643661	*Pgl_GLEAN_10018103*	*wall-associated receptor kinase 2*	Integral component of membrane [GO:0016021]; plasma membrane [GO:0005886]; ATP binding [GO:0005524]; calcium ion binding [GO:0005509]; polysaccharide binding [GO:0030247]; protein serine/threonine kinase activity [GO:0004674]; cell surface receptor signaling pathway [GO:0007166]; protein phosphorylation [GO:0006468]integral component of membrane [GO:0016021]; plasma membrane [GO:0005886]
32	*PMSnp856*	T/C	2	242643661	*Pgl_GLEAN_10018119*	*BAG family molecular chaperone regulator 7*	Chaperone binding [GO:0051087]chaperone binding [GO:0051087]
33	*PMSnpB755*	A/G	3	9505041	*Pgl_GLEAN_10031625*	*Growth-regulating factor*	Nucleus [GO:0005634]; ATP binding [GO:0005524]; developmental process [GO:0032502]; regulation of transcription, DNA-templated [GO:0006355]; transcription, DNA-templated [GO:0006351]nucleus [GO:0005634]

34	*PMSnp949*	A/G	3	47291104	*Pgl_GLEAN_10027272*	*glutaredoxin-C6*	Electron transfer activity [GO:0009055]; protein disulfide oxidoreductase activity [GO:0015035]DNA binding [GO:0003677]; DNA-binding transcription factor activity [GO:0003700]
35	*PMSnp965*	A/G	3	55164251	*Pgl_GLEAN_10025646*	*BAG family molecular chaperone regulator 1*	Chaperone binding [GO:0051087]chaperone binding [GO:0051087]
36	*PMSnp969*	G/A	3	58937590	*Pgl_GLEAN_10024418*	*14-3-3-like protein GF14-E*	Response to cold [GO:0009409]plant-type cell wall [GO:0009505]Protein binding [GO:0005515]brassinosteroid mediated signaling pathway [GO:0009742]
37	*PMSnp1007*	T/A	3	89205847	*Pgl_GLEAN_10036819*	*protein trichome birefringence-like 24*	Membrane [GO:0016020] O-acetyltransferase activity [GO:0016413]
38	*PMSnp1010*	T/A	3	96226449	*Pgl_GLEAN_10032749*	*phosphatidylinositol 4-kinase gamma 4*	Integral component of membrane [GO:0016021]response to salt stress [GO:0009651]; cellular response to hypoxia [GO:0071456]ATP binding [GO:0005524]; kinase activity [GO:0016301]
39	*PMSnp1090*	A/G	3	196802373	*Pgl_GLEAN_10037948*	*homeobox-leucine zipper protein ROC6-like*	Nucleus [GO:0005634]regulation of DNA-templated transcription [GO:0006355]DNA-binding transcription factor activity [GO:0003677], RNA polymerase II-specific [GO:0000981]
40	*PMSnpB1012*	A/G	3	295848019	*Pgl_GLEAN_10003545*	*AP2-like ethylene-responsive transcription factor TOE3*	Nucleus [GO:0005634]; DNA binding [GO:0003677]; DNA-binding transcription factor activity [GO:0003700]ethylene-activated signaling pathway [GO:0009873]protein binding [GO:0005515]
41	*PMSnpB1012*	A/G	3	295848019	*Pgl_GLEAN_10027179*	*serine/threonine-protein kinase GRIK1*	Nucleus [GO:0005634]; cytoplasm [GO:0005737]protein autophosphorylation [GO:0005737]; response to virus [GO:0009615]protein phosphorylation [GO:0006468]; ATP binding [GO:0005524]
42	*PMSnp1255*	A/T	3	296507853	*Pgl_GLEAN_10003545*	*AP2-like ethylene-responsive transcription factor TOE3*	Nucleus [GO:0005634]; DNA binding [GO:0003677]; DNA-binding transcription factor activity [GO:0003700]ethylene-activated signaling pathway [GO:0009873]protein binding [GO:0005515]
43	*PMSnpB1066*	T/A	4	7343447	*Pgl_GLEAN_10012902*	*NLR-encoding gene- ADR1-Like 3*	Cell surface receptor signaling pathway[GO:0007166]; defense response to fungus [GO:0050832]ATP binding [GO:0005524]
44	*PMSnpB1088*	C/T	4	19381035	*Pgl_GLEAN_10032560*	*putative cysteine-rich receptor-like protein kinase 35*	Integral component of membrane [GO:0016020]protein phosphorylation [GO:0006468]protein serine/threonine kinase activity[GO:0004674]; ATP binding [GO:0005524]
45	*PMSnp1322*	C/T	4	20664322	*Pgl_GLEAN_10032560*	*putative cysteine-rich receptor-like protein kinase 35*	Integral component of membrane [GO:0016020]protein phosphorylation [GO:0006468]protein serine/threonine kinase activity[GO:0004674]; ATP binding [GO:0005524]
46	*PMSnpB1100*	G/A	4	21482134	*Pgl_GLEAN_10032560*	*putative cysteine-rich receptor-like protein kinase 35*	Integral component of membrane [GO:0016020]protein phosphorylation [GO:0006468]protein serine/threonine kinase activity[GO:0004674]; ATP binding [GO:0005524]

47	*PMSnpB1100*	G/A	4	21482134	*Pgl_GLEAN_10025439*	*transcription factor MYB83*	Nucleus [GO:0005634]regulation of secondary cell wall biogenesis [GO:2000652]electron transfer activity [GO:0009055]; protein disulfide oxidoreductase activity [GO:0015035]
48	*PMSnp1400*	G/A	4	62242030	*Pgl_GLEAN_10025268*	*protein NRT1/ PtRNAFAMILY 6.3*	Integral component of membrane [GO:0016020]auxin-activated signaling pathway[GO:0009734]; nitrate assimilation[GO:0042128]transmembrane transporter activity [GO:0022857]; protein binding[GO:0005515]
49	*PMSnp1400*	G/A	4	62242030	*Pgl_GLEAN_10023930*	*caffeic acid 3-O-methyltransferase*	Caffeate O-methyltransferase activity [GO:0047763]; protein dimerization activity [GO:0046983]; methylation [GO:0032259]UDP-glycosyltransferase activity [GO:0008194]
50	*PMSnpB1336*	T/G	5	62775477	*Pgl_GLEAN_10005235*	*Protein kinase domain-containing protein*	Integral component of membrane [GO:0016021]; plasma membrane [GO:0005886]; ATP binding [GO:0005524]; protein serine/threonine kinase activity [GO:0004674]; protein phosphorylation [GO:0006468]integral component of membrane [GO:0016021]; plasma membrane [GO:0005886]regulation of transcription, DNA-templated [GO:0006355]protein dimerization activity [GO:0046983]
51	*PMSnpB1484*	A/G	5	127307938	*Pgl_GLEAN_10026157*	*WRKY domain-containing protein*	Nucleus [GO:0005634]; DNA-binding transcription factor activity [GO:0003700]; sequence-specific DNA binding [GO:0043565]nucleus [GO:0005634]ethylene-activated signaling pathway [GO:0009873]; cellular response to phosphate starvation [GO:0016036]UDP-glycosyltransferase activity [GO:0008194]
52	*PMSnp1826*	G/T	5	132834219	*Pgl_GLEAN_10025423*	*L-ascorbate peroxidase*	Heme binding [GO:0020037]; L-ascorbate peroxidase activity [GO:0016688]; metal ion binding [GO:0046872]; response to oxidative stress [GO:0006979]DNA-binding transcription factor activity [GO:0003700]; sequence-specific DNA binding [GO:0043565]
53	*PMSnp1871*	C/T	5	146370002	*Pgl_GLEAN_10011029*	*calmodulin-binding transcription activator 3*	Nucleus [GO:0005634]; calmodulin binding [GO:0005516]; DNA binding [GO:0003677]nucleus [GO:0005634]defense response to fungus [GO:0050832]; defense response to bacterium [GO:0042742]; response to cold [GO:0009409]
54	*PMSnp1894*	T/G	5	151351405	*Pgl_GLEAN_10009612*	*Kinesin-like protein*	Microtubule [GO:0005874]; ATP binding [GO:0005524]; microtubule binding [GO:0008017]; microtubule motor activity [GO:0003777]; microtubule-based movement [GO:0007018]microtubule [GO:0005874]ATP binding [GO:0005524]; protein kinase activity [GO:0004672]
55	*PMSnp1944*	C/T	6	15371005	*Pgl_GLEAN_10029753*	*E3 ubiquitin-protein ligase At3g02290*	Cytoplasm [GO:0005737]response to auxin [GO:0009733]; protein ubiquitination [GO:0016567]
56	*PMSnp1964*	A/G	6	25269605	*Pgl_GLEAN_10031689*	*Patatin*	Hydrolase activity [GO:0016787]; lipid catabolic process [GO:0016042]signal transduction [GO:0007165]protein phosphatase regulator activity [GO:0019888]

57	*PMSnpB1692*	A/C	6	33757181	*Pgl_GLEAN_10004402*	*WRKY domain-containing protein*	Nucleus [GO:0005634]; DNA-binding transcription factor activity [GO:0003700]; sequence-specific DNA binding [GO:0043565]nucleus [GO:0005634]ethylene-activated signaling pathway [GO:0009873]; cellular response to phosphate starvation [GO:0016036]UDP-glycosyltransferase activity [GO:0008194]
58	*PMSnp2014*	C/T	6	56155469	*Pgl_GLEAN_10029246*	*Glutathione transferase*	Glutathione transferase activity [GO:0004364]; glutathione metabolic process [GO:0006749]DNA binding [GO:0003677]; DNA-binding transcription factor activity [GO:0003700]
59	*PMSnp2021*	A/G	6	58789904	*Pgl_GLEAN_10035861*	*Auxin response factor*	Nucleus [GO:0005634]; DNA binding [GO:0003677]; auxin-activated signaling pathway [GO:0009734]; regulation of transcription, DNA-templated [GO:0006355]nucleus [GO:0005634]auxin-activated signaling pathway [GO:0009734]; regulation of transcription, DNA-templated [GO:0006355]DNA binding [GO:0003677]
60	*PMSnpB1733*	A/G	6	58931372	*Pgl_GLEAN_10035861*	*Auxin response factor*	Nucleus [GO:0005634]; DNA binding [GO:0003677]; auxin-activated signaling pathway [GO:0009734]; regulation of transcription, DNA-templated [GO:0006355]nucleus [GO:0005634]auxin-activated signaling pathway [GO:0009734]; regulation of transcription, DNA-templated [GO:0006355]DNA binding [GO:0003677]
61	*PMSnp2023*	T/C	6	58989110	*Pgl_GLEAN_10035861*	*Auxin response factor*	Nucleus [GO:0005634]; DNA binding [GO:0003677]; auxin-activated signaling pathway [GO:0009734]; regulation of transcription, DNA-templated [GO:0006355]nucleus [GO:0005634]auxin-activated signaling pathway [GO:0009734]; regulation of transcription, DNA-templated [GO:0006355]DNA binding [GO:0003677]
62	*PMSnp2031*	T/G	6	62256368	*Pgl_GLEAN_10010984*	*BURP domain-containing protein*	Regulation of transcription, DNA-templated [GO:0006355]calmodulin binding [GO:0005516]; DNA binding [GO:0003677]; zinc ion binding [GO:0008270]
63	*PMSnpB1739*	T/A	6	64552763	*Pgl_GLEAN_10037339*	*transcription factor MYB4*	Nucleus [GO:0005634]regulation of phenylpropanoid metabolic process [GO:2000762]protein binding [GO:0005515]; DNA-binding transcription factor activity [GO:0003700]
64	*PMSnpB1749*	T/A	6	79961283	*Pgl_GLEAN_10032505*	*Serine/threonine protein phosphatase*	Protein phosphatase type 2A complex [GO:0000159]; protein phosphatase regulator activity [GO:0019888]; signal transduction [GO:0007165]protein phosphatase type 2A complex [GO:0000159]cellulose biosynthetic process [GO:0030244]; cell wall organization [GO:0071555]cellulose synthase (UDP-forming) activity [GO:0016760]; metal ion binding [GO:0046872]
65	*PMSnp2073*	A/G	6	94716225	*Pgl_GLEAN_10020844*	*CASP-like protein*	Integral component of membrane [GO:0016021]; plasma membrane [GO:0005886]integral component of membrane

							[GO:0016021]; plasma membrane [GO:0005886]lipid catabolic process [GO:0016042]hydrolase activity [GO:0016787]
66	*PMSnp2222*	T/C	6	221883066	*Pgl_GLEAN_10019061*	*probable LRR receptor-like kinase At3g47570*	Plasma membrane [GO:0005886]chromatin remodeling [GO:0006338]; protein phosphorylation [GO:0006468]protein serine/threonine kinase activity [GO:0004674]; ATP binding [GO:0005524]; protein kinase activity [GO:0004672]
67	*PMSnpB1925*	T/C	7	6040422	*Pgl_GLEAN_10024314*	*NBS-LRR-like protein*	ADP binding [GO:0043531]; ATP binding [GO:0005524]; defense response [GO:0006952]hydrogen peroxide catabolic process [GO:0042744]; response to oxidative stress [GO:0006979]heme binding [GO:0020037]; metal ion binding [GO:0046872]; peroxidase activity [GO:0004601]
68	*PMSnp2330*	G/T	7	22779127	*Pgl_GLEAN_10031009*	*Receptor-like serine/threonine-protein*	Integral component of membrane [GO:0016021]; ATP binding [GO:0005524]; protein serine kinase activity [GO:0106310]; protein threonine kinase activity [GO:0106311]; recognition of pollen [GO:0048544]integral component of membrane [GO:0016021]lipid catabolic process [GO:0016042]hydrolase activity [GO:0016787]
69	*PMSnp2330*	G/T	7	22779127	*Pgl_GLEAN_10008778*	*Glutaredoxin domain-containing protein*	Chloroplast stroma [GO:0009570]; mitochondrial matrix [GO:0005759]; mitochondrion [GO:0005739]; iron-sulfur cluster binding [GO:0051536]; metal ion binding [GO:0046872]; protein disulfide oxidoreductase activity [GO:0015035]; cation transport [GO:0006812]chloroplast stroma [GO:0009570]; mitochondrial matrix [GO:0005759]; mitochondrion [GO:0005739]
70	*PMSnp2356*	A/G	7	30152925	*Pgl_GLEAN_10024350*	*Glycosyltransferase*	UDP-glycosyltransferase activity [GO:0008194]response to oxidative stress [GO:0006979]; heme binding [GO:0020037]; L-ascorbate peroxidase activity [GO:0016688]; metal ion binding [GO:0046872]
71	*PMSnpB1995*	A/G	7	42783318	*Pgl_GLEAN_10030761*	*auxin-responsive protein SAUR36*	Response to auxin [GO:0009733]developmental process [GO:0032502]; regulation of transcription, DNA-templated [GO:0006355]; transcription, DNA-templated [GO:0006351]ATP binding [GO:0005524]
72	*PMSnp2402*	C/G	7	51192172	*Pgl_GLEAN_10007598*	*BHLH domain-containing protein*	Nucleus [GO:0005634]; protein dimerization activity [GO:0046983]; regulation of transcription, DNA-templated [GO:0006355]nucleus [GO:0005634]microtubule-based movement [GO:0007018]ATP binding [GO:0005524]; microtubule binding [GO:0008017]; microtubule motor activity [GO:0003777]
73	*PMSnpB2031*	A/G	7	83398767	*Pgl_GLEAN_10035106*	*Cellulose synthase*	Integral component of membrane [GO:0016021]; plasma membrane [GO:0005886]; cellulose synthase (UDP-forming) activity [GO:0016760]; metal ion binding [GO:0046872]; cell wall organization [GO:0071555]; cellulose biosynthetic process [GO:0030244]integral component of membrane [GO:0016021]; plasma membrane [GO:0005886]

74	*PMSnp2461*	C/T	7	83517310	*Pgl_GLEAN_10035106*	*Cellulose synthase*	Integral component of membrane [GO:0016021]; plasma membrane [GO:0005886]; cellulose synthase (UDP-forming) activity [GO:0016760]; metal ion binding [GO:0046872]; cell wall organization [GO:0071555]; cellulose biosynthetic process [GO:0030244]integral component of membrane [GO:0016021]; plasma membrane [GO:0005886]
75	*PMSnp2469*	T/G	7	88319524	*Pgl_GLEAN_10028306*	*Glycosyltransferase*	Intracellular membrane-bounded organelle [GO:0043231]; UDP-glycosyltransferase activity [GO:0008194]intracellular membrane-bounded organelle [GO:0043231]ADP binding [GO:0043531]
76	*PMSnp2469*	T/G	7	88319524	*Pgl_GLEAN_10028317*	*AP2/ERF domain-containing protein*	Nucleus [GO:0005634]; cytoplasm [GO:0005737]protein autophosphorylation [GO:0005737]; response to virus [GO:0009615]protein phosphorylation [GO:0006468]; ATP binding [GO:0005524]
77	*PMSnp2575*	T/A	7	151315952	*Pgl_GLEAN_10006309*	*JmjC domain-containing protein*	Chromatin [GO:0000785]; nucleus [GO:0005634]cation transport [GO:0006812]; proteasome-mediated ubiquitin-dependent protein catabolic process [GO:0043161]iron-sulfur cluster binding [GO:0051536]; metal ion binding [GO:0046872]; protein disulfide oxidoreductase activity [GO:0015035]; ubiquitin conjugating enzyme binding [GO:0031624]

Table S2: BLAST results showing gene homology between *Pennisetum glaucum* genes and genes from *Oryza sativa, Zea mays, Setaria italica* and *Sorghum bicolor*.

**Gene_Id (*P. glacum*)**	**Species**	**Chromosome Number**	**Percent similarity**	**Alignment length**	**Mismatch**	**Gap**	**Start**	**End**	**e value**	**bit**
Pgl_GLEAN_10024314	*Oryza sativa*	Oschr11	71.596	1278	285	52	27351827	27353080	2.16E-72	278
Pgl_GLEAN_10024314	*Oryza sativa*	Oschr11	71.821	1274	285	51	27271293	27272542	5.96E-78	296
Pgl_GLEAN_10024314	*Oryza sativa*	Oschr11	72.406	1272	284	50	27343741	27344992	7.54E-92	342
Pgl_GLEAN_10024314	*Oryza sativa*	Oschr11	73.726	1256	260	49	27262931	27264164	2E-117	427
Pgl_GLEAN_10024314	*Oryza sativa*	Oschr11	74.158	1099	231	36	27223696	27224779	2.6E-111	407
Pgl_GLEAN_10024314	*Oryza sativa*	Oschr11	74.171	875	198	23	27244253	27245113	9.76E-91	339
Pgl_GLEAN_10024350	*Oryza sativa*	Oschr7	74.211	1268	256	42	19038142	19039396	7.2E-129	464
Pgl_GLEAN_10019061	*Oryza sativa*	Oschr1	74.586	1086	231	38	2813050	2814115	1.3E-119	435
Pgl_GLEAN_10032749	*Oryza sativa*	Oschr2	74.88	1250	284	23	10996568	10995337	3.9E-152	542
Pgl_GLEAN_10019061	*Oryza sativa*	Oschr1	75.873	630	137	12	2689813	2689192	2.86E-81	307
Pgl_GLEAN_10031625	*Oryza sativa*	Oschr2	76.519	609	77	21	32841550	32840975	3.12E-71	272
Pgl_GLEAN_10031625	*Oryza sativa*	Oschr3	77.562	566	78	26	18694470	18695007	1.85E-78	296
Pgl_GLEAN_10024350	*Oryza sativa*	Oschr7	78.184	1013	189	31	19009152	19008153	5.2E-175	617
Pgl_GLEAN_10018103	*Oryza sativa*	Oschr2	78.331	863	161	20	630464	629612	8.4E-150	534
Pgl_GLEAN_10028688	*Oryza sativa*	Oschr10	79.242	660	108	21	18636096	18635444	9.4E-120	433
Pgl_GLEAN_10036819	*Oryza sativa*	Oschr6	79.513	781	143	16	19417252	19418022	1.1E-151	540
Pgl_GLEAN_10032505	*Oryza sativa*	Oschr5	80.494	1174	216	10	27603032	27604197	0	887
Pgl_GLEAN_10028688	*Oryza sativa*	Oschr10	81.847	639	100	8	18638429	18637792	5.4E-147	523
Pgl_GLEAN_10024350	*Oryza sativa*	Oschr7	82.224	1187	195	11	19061072	19062247	0	1009
Pgl_GLEAN_10031009	*Oryza sativa*	Oschr7	82.727	2472	398	12	22039634	22037167	0	2172
Pgl_GLEAN_10025268	*Oryza sativa*	Oschr10	82.784	819	125	10	21762062	21761246	0	717
Pgl_GLEAN_10018103	*Oryza sativa*	Oschr1	82.835	903	128	17	22456478	22455585	0	784
Pgl_GLEAN_10004433	*Oryza sativa*	Oschr11	83.683	619	93	6	466389	465775	2.4E-162	577
Pgl_GLEAN_10004433	*Oryza sativa*	Oschr12	83.845	619	92	6	544776	544162	5.2E-164	582

Table S2 Continued…

Pgl_GLEAN_10021949	*Oryza sativa*	Oschr6	84.574	564	86	1	3266130	3265567	2.2E-157	558
Pgl_GLEAN_10018073	*Oryza sativa*	Oschr2	84.641	612	86	6	826382	825778	1.5E-170	603
Pgl_GLEAN_10018103	*Oryza sativa*	Oschr2	84.703	1144	165	6	628960	627825	0	1134
Pgl_GLEAN_10018073	*Oryza sativa*	Oschr3	84.75	1082	150	13	1789905	1788833	0	1070
Pgl_GLEAN_10010984	*Oryza sativa*	Oschr1	85.114	1095	140	13	30591170	30592258	0	1098
Pgl_GLEAN_10012902	*Oryza sativa*	Oschr12	85.563	755	107	2	24456850	24457603	0	789
Pgl_GLEAN_10008778	*Oryza sativa*	Oschr12	85.658	509	73	0	3850211	3849703	8.8E-151	536
Pgl_GLEAN_10023930	*Oryza sativa*	Oschr8	87.259	675	83	3	3338716	3338042	0	767
Pgl_GLEAN_10025523	*Oryza sativa*	Oschr1	87.478	567	70	1	13924645	13924079	0	652
Pgl_GLEAN_10012902	*Oryza sativa*	Oschr12	88	850	98	4	24458139	24458985	0	1002
Pgl_GLEAN_10025268	*Oryza sativa*	Oschr8	88.226	603	60	5	3189292	3188691	0	710
Pgl_GLEAN_10018073	*Oryza sativa*	Oschr2	88.409	811	89	4	827271	826465	0	972
Pgl_GLEAN_10035106	*Oryza sativa*	Oschr3	88.927	587	65	0	33781385	33780799	0	725
Pgl_GLEAN_10035106	*Oryza sativa*	Oschr7	91.98	586	47	0	5853641	5853056	0	822
Pgl_GLEAN_10000574	*Zea mays*	ZmChr2	84.481	1089	159	9	233142296	2.33E+08	0	1066
Pgl_GLEAN_10000574	*Zea mays*	ZmChr2	84.89	867	122	7	31424633	31423770	0	867
Pgl_GLEAN_10000574	*Zea mays*	ZmChr2	85.598	861	123	1	65529304	65528445	0	902
Pgl_GLEAN_10004433	*Zea mays*	ZmChr4	83.414	621	79	10	189129657	1.89E+08	6.6E-155	555
Pgl_GLEAN_10004433	*Zea mays*	ZmChr2	84.39	615	72	6	156652440	1.57E+08	3E-163	582
Pgl_GLEAN_10010984	*Zea mays*	ZmChr8	85.835	946	117	10	182261592	1.82E+08	0	989
Pgl_GLEAN_10010984	*Zea mays*	ZmChr3	87.061	1167	115	22	205663460	2.06E+08	0	1286
Pgl_GLEAN_10011010	*Zea mays*	ZmChr6	87.873	503	55	1	165568567	1.66E+08	1.5E-164	586
Pgl_GLEAN_10011029	*Zea mays*	ZmChr5	79.815	649	119	10	39697447	39696805	3.2E-127	462
Pgl_GLEAN_10011029	*Zea mays*	ZmChr1	79.876	646	117	11	142194208	1.42E+08	1.1E-126	460
Pgl_GLEAN_10011029	*Zea mays*	ZmChr1	87.896	694	82	2	19957125	19956433	0	815
Pgl_GLEAN_10011029	*Zea mays*	ZmChr1	89.845	581	59	0	19956361	19955781	0	747
Pgl_GLEAN_10012902	*Zea mays*	ZmChr1	86.605	754	101	0	167989581	1.68E+08	0	833
Pgl_GLEAN_10012902	*Zea mays*	ZmChr1	87.055	842	103	4	167990768	1.68E+08	0	946
Pgl_GLEAN_10012902	*Zea mays*	ZmChr3	90.55	836	79	0	114690495	1.15E+08	0	1107
Pgl_GLEAN_10017120	*Zea mays*	ZmChr4	86.285	576	66	10	4576900	4577464	1.5E-173	614

Pgl_GLEAN_10018073	*Zea mays*	ZmChr1	81.437	1169	193	22	7099560	7098404	0	935
Pgl_GLEAN_10018073	*Zea mays*	ZmChr4	90.141	1420	131	8	248819954	2.49E+08	0	1838
Pgl_GLEAN_10018178	*Zea mays*	ZmChr5	85.464	571	72	5	72857985	72857425	3.8E-164	584
Pgl_GLEAN_10019061	*Zea mays*	ZmChr8	76.42	581	117	15	15297025	15296446	3.6E-77	296
Pgl_GLEAN_10020844	*Zea mays*	ZmChr3	81.339	702	107	10	217579473	2.18E+08	8.6E-154	549
Pgl_GLEAN_10021949	*Zea mays*	ZmChr9	85.589	569	81	1	18135883	18136451	9.5E-168	595
Pgl_GLEAN_10021949	*Zea mays*	ZmChr6	87.873	569	68	1	88380335	88379767	0	667
Pgl_GLEAN_10023930	*Zea mays*	ZmChr4	89.349	676	68	3	34199815	34199142	0	846
Pgl_GLEAN_10024350	*Zea mays*	ZmChr7	74.98	1227	262	37	160105744	1.6E+08	6.8E-146	523
Pgl_GLEAN_10024350	*Zea mays*	ZmChr7	76.446	1210	247	34	160109300	1.6E+08	2.4E-175	621
Pgl_GLEAN_10024350	*Zea mays*	ZmChr2	77.764	796	146	29	240983148	2.41E+08	5.4E-127	460
Pgl_GLEAN_10024350	*Zea mays*	ZmChr9	84.669	1435	182	21	19062195	19060765	0	1397
Pgl_GLEAN_10025268	*Zea mays*	ZmChr10	87.007	608	64	8	81343636	81344243	0	671
Pgl_GLEAN_10025523	*Zea mays*	ZmChr8	86.323	563	77	0	41234479	41235041	2.6E-173	614
Pgl_GLEAN_10025523	*Zea mays*	ZmChr6	89.965	568	56	1	138000357	1.38E+08	0	732
Pgl_GLEAN_10025523	*Zea mays*	ZmChr8	91.901	568	45	1	84800069	84800636	0	793
Pgl_GLEAN_10028542	*Zea mays*	ZmChr2	77.636	626	122	10	238732595	2.39E+08	7.64E-98	364
Pgl_GLEAN_10031009	*Zea mays*	ZmChr7	87.576	2463	279	10	165448849	1.65E+08	0	2828
Pgl_GLEAN_10031625	*Zea mays*	ZmChr4	80.808	594	75	15	182169123	1.82E+08	1E-117	429
Pgl_GLEAN_10032749	*Zea mays*	ZmChr9	86.024	1338	184	1	56917609	56916275	0	1432
Pgl_GLEAN_10032749	*Zea mays*	ZmChr6	87.145	1338	169	1	117768320	1.18E+08	0	1515
Pgl_GLEAN_10033865	*Zea mays*	ZmChr1	80.458	568	72	16	274141048	2.74E+08	3.8E-108	398
Pgl_GLEAN_10033865	*Zea mays*	ZmChr4	80.708	565	67	18	228446728	2.28E+08	2.9E-109	401
Pgl_GLEAN_10033865	*Zea mays*	ZmChr1	81.658	567	62	19	12555911	12556450	1E-118	433
Pgl_GLEAN_10033865	*Zea mays*	ZmChr5	81.944	576	59	25	5411806	5411261	1.3E-122	446
Pgl_GLEAN_10033865	*Zea mays*	ZmChr8	81.947	565	60	18	8350790	8351327	6.2E-121	440
Pgl_GLEAN_10033865	*Zea mays*	ZmChr1	82.812	576	54	19	290411826	2.9E+08	6.1E-131	473
Pgl_GLEAN_10035106	*Zea mays*	ZmChr7	83.986	587	94	0	19135787	19135201	9.1E-158	564
Pgl_GLEAN_10035106	*Zea mays*	ZmChr1	85.034	588	86	2	297611905	2.98E+08	9E-168	597
Pgl_GLEAN_10035106	*Zea mays*	ZmChr2	86.031	587	82	0	165517437	1.66E+08	8.9E-178	630

Pgl_GLEAN_10035861	*Zea mays*	ZmChr8	83.878	887	134	7	180712877	1.81E+08	0	837
Pgl_GLEAN_10035861	*Zea mays*	ZmChr3	84.004	894	129	14	201735869	2.02E+08	0	846
Pgl_GLEAN_10025268	*Setaria italica*	Sichr1	74.154	739	145	35	28913020	28912299	1.08E-68	265
Pgl_GLEAN_10019061	*Setaria italica*	Sichr5	74.163	1583	367	34	9074872	9076434	3.5E-175	619
Pgl_GLEAN_10024314	*Setaria italica*	Sichr8	74.334	2552	576	58	29492894	29495399	0	1011
Pgl_GLEAN_10024350	*Setaria italica*	Sichr2	74.434	1236	259	49	41234567	41235784	2.8E-133	479
Pgl_GLEAN_10024350	*Setaria italica*	Sichr2	74.48	1203	231	64	41255968	41257129	6.1E-125	451
Pgl_GLEAN_10036899	*Setaria italica*	Sichr4	75.114	876	180	23	14142250	14143112	7.1E-102	375
Pgl_GLEAN_10036899	*Setaria italica*	Sichr4	75.957	836	168	20	14142274	14143100	4.2E-109	399
Pgl_GLEAN_10036899	*Setaria italica*	Sichr4	76.257	935	185	24	14142249	14143169	5.3E-128	462
Pgl_GLEAN_10019061	*Setaria italica*	Sichr5	76.371	1003	211	21	9068701	9069691	4.7E-144	516
Pgl_GLEAN_10024350	*Setaria italica*	Sichr2	76.72	1366	275	35	41220883	41222225	0	721
Pgl_GLEAN_10036899	*Setaria italica*	Sichr4	76.823	919	180	23	14142252	14143160	3.2E-135	486
Pgl_GLEAN_10036899	*Setaria italica*	Sichr4	77.322	926	188	16	14142249	14143172	1.9E-147	527
Pgl_GLEAN_10024314	*Setaria italica*	Sichr8	77.419	1426	287	21	38451101	38452516	0	817
Pgl_GLEAN_10036899	*Setaria italica*	Sichr4	77.674	748	146	12	14142431	14143172	3.2E-120	436
Pgl_GLEAN_10036899	*Setaria italica*	Sichr4	77.921	933	172	20	14142251	14143172	1.1E-154	551
Pgl_GLEAN_10036899	*Setaria italica*	Sichr4	77.948	848	163	13	14142331	14143172	6.7E-142	508
Pgl_GLEAN_10024314	*Setaria italica*	Sichr8	78.638	1395	286	12	29532901	29534289	0	915
Pgl_GLEAN_10025268	*Setaria italica*	Sichr9	79.198	524	88	15	38241330	38241847	1.35E-92	344
Pgl_GLEAN_10019061	*Setaria italica*	Sichr5	79.643	2240	437	16	9064455	9066683	0	1592
Pgl_GLEAN_10028688	*Setaria italica*	Sichr9	80.344	697	111	19	15036716	15036033	2.1E-141	505
Pgl_GLEAN_10024314	*Setaria italica*	Sichr3	80.512	1406	267	4	16150823	16149420	0	1072
Pgl_GLEAN_10028688	*Setaria italica*	Sichr9	80.727	633	102	16	15053991	15053361	1.7E-132	475
Pgl_GLEAN_10032505	*Setaria italica*	Sichr3	81.356	1003	176	8	11124239	11123244	0	806
Pgl_GLEAN_10010984	*Setaria italica*	Sichr5	82.156	538	71	9	36056918	36057443	4.7E-121	438
Pgl_GLEAN_10036899	*Setaria italica*	Sichr4	82.397	1744	260	35	14142246	14143964	0	1476
Pgl_GLEAN_10018103	*Setaria italica*	Sichr1	83.258	890	135	11	10358388	10359269	0	806
Pgl_GLEAN_10025439	*Setaria italica*	Sichr3	83.993	606	33	22	45042884	45043436	1.2E-146	523
Pgl_GLEAN_10019061	*Setaria italica*	Sichr5	84.277	954	133	13	8925588	8926537	0	915

Pgl_GLEAN_10018073	*Setaria italica*	Sichr9	84.538	1093	155	12	57026636	57027720	0	1070
Pgl_GLEAN_10019061	*Setaria italica*	Sichr5	84.615	949	136	7	8863799	8864745	0	935
Pgl_GLEAN_10019061	*Setaria italica*	Sichr5	84.868	945	134	6	8982360	8983301	0	944
Pgl_GLEAN_10025523	*Setaria italica*	Sichr5	85.714	567	80	1	15499619	15499053	4.9E-169	597
Pgl_GLEAN_10024350	*Setaria italica*	Sichr2	86.473	1449	179	10	41252279	41253714	0	1574
Pgl_GLEAN_10010984	*Setaria italica*	Sichr2	87.022	732	79	12	31907529	31906808	0	811
Pgl_GLEAN_10035106	*Setaria italica*	Sichr9	88.245	587	69	0	2479831	2480417	0	702
Pgl_GLEAN_10028317	*Setaria italica*	Sichr2	88.649	925	79	9	6489822	6490730	0	1103
Pgl_GLEAN_10028306	*Setaria italica*	Sichr2	88.676	574	41	4	6337907	6338480	0	680
Pgl_GLEAN_10023779	*Setaria italica*	Sichr8	89.282	1726	131	27	29295644	29297330	0	2113
Pgl_GLEAN_10028306	*Setaria italica*	Sichr2	89.635	521	48	4	6348823	6349337	0	658
Pgl_GLEAN_10037948	*Setaria italica*	Sichr7	89.925	536	54	0	16179076	16178541	0	691
Pgl_GLEAN_10037948	*Setaria italica*	Sichr8	90.056	533	53	0	6203119	6203651	0	691
Pgl_GLEAN_10028306	*Setaria italica*	Sichr2	90.244	533	46	5	6337045	6337571	0	691
Pgl_GLEAN_10000574	*Setaria italica*	Sichr8	90.406	1084	104	0	30522985	30521902	0	1426
Pgl_GLEAN_10031625	*Setaria italica*	Sichr1	90.576	573	27	12	39561877	39561332	0	734
Pgl_GLEAN_10036819	*Setaria italica*	Sichr4	90.677	783	71	2	26162985	26163766	0	1040
Pgl_GLEAN_10023779	*Setaria italica*	Sichr8	91.723	894	74	0	29297656	29298549	0	1242
Pgl_GLEAN_10033865	*Setaria italica*	Sichr9	92.255	581	27	1	3909913	3909333	0	808
Pgl_GLEAN_10020844	*Setaria italica*	Sichr5	92.389	565	31	3	31597902	31597344	0	795
Pgl_GLEAN_10000574	*Setaria italica*	Sichr1	92.435	1084	82	0	4637886	4638969	0	1548
Pgl_GLEAN_10001581	*Setaria italica*	Sichr3	92.5	520	27	5	680721	680214	0	734
Pgl_GLEAN_10018178	*Setaria italica*	Sichr1	92.768	719	28	13	9658642	9657947	0	1018
Pgl_GLEAN_10018073	*Setaria italica*	Sichr1	93.155	1417	91	1	10243352	10241942	0	2074
Pgl_GLEAN_10010984	*Setaria italica*	Sichr5	94.171	1218	62	6	36056977	36058191	0	1847
Pgl_GLEAN_10018103	*Setaria italica*	Sichr1	94.435	1168	59	1	10360638	10361799	0	1792
Pgl_GLEAN_10031009	*Setaria italica*	Sichr2	94.505	2475	115	6	42938039	42935565	0	3797
Pgl_GLEAN_10017120	*Setaria italica*	Sichr8	94.792	576	27	2	36943183	36942611	0	894
Pgl_GLEAN_10032749	*Setaria italica*	Sichr4	94.847	1339	67	2	23946132	23947469	0	2089
Pgl_GLEAN_10023930	*Setaria italica*	Sichr6	95.104	674	33	0	4464221	4464894	0	1062

Table S2 Continued…

Pgl_GLEAN_10032505	*Setaria italica*	Sichr5	95.21	1169	56	0	33421893	33420725	0	1849
Pgl_GLEAN_10011029	*Setaria italica*	Sichr9	95.382	693	32	0	54241339	54242031	0	1103
Pgl_GLEAN_10035861	*Setaria italica*	Sichr5	95.8	881	37	0	37047726	37046846	0	1423
Pgl_GLEAN_10025268	*Setaria italica*	Sichr6	96.121	593	23	0	4735340	4735932	0	968
Pgl_GLEAN_10012902	*Setaria italica*	Sichr3	96.154	754	29	0	47383956	47384709	0	1232
Pgl_GLEAN_10011029	*Setaria italica*	Sichr9	96.558	581	20	0	54242099	54242679	0	963
Pgl_GLEAN_10008778	*Setaria italica*	Sichr2	96.857	509	16	0	43103712	43104220	0	852
Pgl_GLEAN_10012902	*Setaria italica*	Sichr3	96.89	836	26	0	47385360	47386195	0	1400
Pgl_GLEAN_10021949	*Setaria italica*	Sichr4	96.98	563	17	0	4572702	4573264	0	946
Pgl_GLEAN_10035106	*Setaria italica*	Sichr2	97.104	587	17	0	6997633	6997047	0	990
Pgl_GLEAN_10025523	*Setaria italica*	Sichr7	97.203	572	14	2	31929087	31929658	0	966
Pgl_GLEAN_10004433	*Setaria italica*	Sichr8	97.236	615	17	0	960027	960641	0	1042
Pgl_GLEAN_10004433	*Setaria italica*	Sichr7	97.724	615	14	0	35590578	35591192	0	1059
Pgl_GLEAN_10004433	*Setaria italica*	Sichr7	97.724	615	14	0	35617502	35618116	0	1059
Pgl_GLEAN_10024350	*Sorghum bicolor*	Sbchr2	73.744	1154	246	46	68567846	68568978	3.9E-109	399
Pgl_GLEAN_10019061	*Sorghum bicolor*	Sbchr3	74.226	1583	346	50	5738028	5736479	4.8E-171	606
Pgl_GLEAN_10024350	*Sorghum bicolor*	Sbchr6	75.228	1207	256	34	48967949	48966760	3.7E-149	532
Pgl_GLEAN_10019061	*Sorghum bicolor*	Sbchr3	75.528	2558	589	34	6000802	5998264	0	1221
Pgl_GLEAN_10024350	*Sorghum bicolor*	Sbchr2	75.618	1214	256	35	68538249	68539442	3.6E-159	566
Pgl_GLEAN_10019061	*Sorghum bicolor*	Sbchr3	75.627	2790	611	56	5992702	5989948	0	1321
Pgl_GLEAN_10024350	*Sorghum bicolor*	Sbchr2	76.379	1215	251	32	68530910	68532104	7.7E-176	621
Pgl_GLEAN_10024350	*Sorghum bicolor*	Sbchr2	76.514	1073	206	38	68513318	68512263	1.7E-152	544
Pgl_GLEAN_10024314	*Sorghum bicolor*	Sbchr5	77.799	1072	227	11	69878999	69880066	0	651
Pgl_GLEAN_10024350	*Sorghum bicolor*	Sbchr2	78.152	1396	251	38	68561323	68562688	0	839
Pgl_GLEAN_10025268	*Sorghum bicolor*	Sbchr1	78.479	539	98	15	58609061	58609593	3.96E-90	337
Pgl_GLEAN_10025439	*Sorghum bicolor*	Sbchr8	78.912	588	75	28	51884682	51884133	2.85E-95	353
Pgl_GLEAN_10019061	*Sorghum bicolor*	Sbchr3	79.072	2781	512	45	5951467	5948718	0	1847
Pgl_GLEAN_10011029	*Sorghum bicolor*	Sbchr1	79.724	651	120	9	36160374	36161018	3.7E-127	460
Pgl_GLEAN_10020844	*Sorghum bicolor*	Sbchr3	79.82	887	114	30	58483925	58484777	2.1E-165	586
Pgl_GLEAN_10019061	*Sorghum bicolor*	Sbchr3	79.868	606	118	3	5770835	5770232	5.1E-121	440

Pgl_GLEAN_10025268	*Sorghum bicolor*	Sbchr1	80.649	770	125	22	80513218	80512458	7.7E-162	575
Pgl_GLEAN_10028317	*Sorghum bicolor*	Sbchr2	80.972	967	116	31	7249517	7250467	0	704
Pgl_GLEAN_10018178	*Sorghum bicolor*	Sbchr4	81.806	742	98	20	1339014	1338286	9.6E-166	588
Pgl_GLEAN_10022624	*Sorghum bicolor*	Sbchr1	81.98	505	76	6	27878157	27877653	1.2E-113	414
Pgl_GLEAN_10018103	*Sorghum bicolor*	Sbchr6	82.014	884	148	7	58241016	58240137	0	741
Pgl_GLEAN_10018073	*Sorghum bicolor*	Sbchr1	82.733	1083	172	13	78384995	78386068	0	950
Pgl_GLEAN_10024350	*Sorghum bicolor*	Sbchr10	83.925	1437	196	20	3771016	3769581	0	1341
Pgl_GLEAN_10031625	*Sorghum bicolor*	Sbchr4	84.22	583	54	12	65268012	65267458	2.5E-149	532
Pgl_GLEAN_10000574	*Sorghum bicolor*	Sbchr3	84.467	1088	161	7	72517846	72518929	0	1066
Pgl_GLEAN_10035106	*Sorghum bicolor*	Sbchr1	84.79	572	83	3	3385908	3386477	1.8E-160	571
Pgl_GLEAN_10024350	*Sorghum bicolor*	Sbchr10	85	1440	181	22	3763803	3765238	0	1430
Pgl_GLEAN_10035861	*Sorghum bicolor*	Sbchr3	85.844	883	121	4	62994484	62993604	0	935
Pgl_GLEAN_10025523	*Sorghum bicolor*	Sbchr3	86.067	567	78	1	18174289	18173723	3.9E-172	608
Pgl_GLEAN_10004433	*Sorghum bicolor*	Sbchr8	86.473	621	60	10	782112	781516	0	660
Pgl_GLEAN_10021949	*Sorghum bicolor*	Sbchr10	86.643	569	75	1	4008872	4008304	3.1E-178	628
Pgl_GLEAN_10018103	*Sorghum bicolor*	Sbchr4	86.773	877	116	0	722484	721608	0	977
Pgl_GLEAN_10032749	*Sorghum bicolor*	Sbchr10	87.818	1338	160	1	41298917	41300251	0	1565
Pgl_GLEAN_10031009	*Sorghum bicolor*	Sbchr2	87.897	2454	267	10	70308064	70305620	0	2859
Pgl_GLEAN_10011029	*Sorghum bicolor*	Sbchr1	88.023	693	83	0	74676488	74677180	0	821
Pgl_GLEAN_10010984	*Sorghum bicolor*	Sbchr3	88.05	1364	139	10	62010770	62012133	0	1594
Pgl_GLEAN_10025268	*Sorghum bicolor*	Sbchr7	88.648	599	62	2	4495733	4496331	0	725
Pgl_GLEAN_10011010	*Sorghum bicolor*	Sbchr9	88.889	504	48	4	53087464	53087960	2.2E-173	614
Pgl_GLEAN_10032505	*Sorghum bicolor*	Sbchr3	89.222	1169	126	0	59723359	59722191	0	1461
Pgl_GLEAN_10012902	*Sorghum bicolor*	Sbchr8	89.474	836	88	0	58032158	58032993	0	1057
Pgl_GLEAN_10023930	*Sorghum bicolor*	Sbchr7	89.956	677	59	6	4722232	4721557	0	865
Pgl_GLEAN_10011029	*Sorghum bicolor*	Sbchr1	90.361	581	56	0	74677254	74677834	0	763
Pgl_GLEAN_10025523	*Sorghum bicolor*	Sbchr9	90.493	568	53	1	5001993	5002560	0	749
Pgl_GLEAN_10018103	*Sorghum bicolor*	Sbchr4	91.104	1169	96	3	720254	719093	0	1576
Pgl_GLEAN_10018073	*Sorghum bicolor*	Sbchr4	91.197	1420	113	8	949866	948456	0	1919
Pgl_GLEAN_10012902	*Sorghum bicolor*	Sbchr8	91.379	754	65	0	58030921	58031674	0	1033
Pgl_GLEAN_10035106	*Sorghum bicolor*	Sbchr2	92.164	587	46	0	7813568	7812982	0	830
Pgl_GLEAN_10008778	*Sorghum bicolor*	Sbchr5	92.731	509	37	0	235025	234517	0	736

Table S3: List of inbreds used in the study

Sl. No	Inbred ID	Group	Sl. No	Inbred ID	Group	Sl. No	Inbred ID	Group	Sl. No	Inbred ID	Group	Sl. No	Inbred ID	Group	Sl. No	Inbred ID	Group
1	TCB1	B line	51	TCB153	B line	101	TCB29	B line	151	TCB95	B line	201	TCR150	R line	251	TCR62	R line
2	TCB10	B line	52	TCB154	B line	102	TCB3	B line	152	TCB96	B line	202	TCR151	R line	252	TCR63	R line
3	TCB100	B line	53	TCB155	B line	103	TCB31	B line	153	TCB97	B line	203	TCR153	R line	253	TCR64	R line
4	TCB101	B line	54	TCB156	B line	104	TCB32	B line	154	TCB98	B line	204	TCR157	R line	254	TCR65	R line
5	TCB102	B line	55	TCB157	B line	105	TCB33	B line	155	TCR1	R line	205	TCR158	R line	255	TCR66	R line
6	TCB103	B line	56	TCB158	B line	106	TCB34	B line	156	TCR10	R line	206	TCR159	R line	256	TCR67	R line
7	TCB104	B line	57	TCB159	B line	107	TCB4	B line	157	TCR100	R line	207	TCR16	R line	257	TCR68	R line
8	TCB105	B line	58	TCB160	B line	108	TCB40	B line	158	TCR101	R line	208	TCR160	R line	258	TCR69	R line
9	TCB106	B line	59	TCB161	B line	109	TCB41	B line	159	TCR102	R line	209	TCR161	R line	259	TCR7	R line
10	TCB108	B line	60	TCB162	B line	110	TCB42	B line	160	TCR103	R line	210	TCR162	R line	260	TCR72	R line
11	TCB110	B line	61	TCB163	B line	111	TCB43	B line	161	TCR105	R line	211	TCR167	R line	261	TCR75	R line
12	TCB111	B line	62	TCB165	B line	112	TCB45	B line	162	TCR107	R line	212	TCR168	R line	262	TCR76	R line
13	TCB112	B line	63	TCB166	B line	113	TCB46	B line	163	TCR108	R line	213	TCR169	R line	263	TCR77	R line
14	TCB113	B line	64	TCB167	B line	114	TCB47	B line	164	TCR11	R line	214	TCR17	R line	264	TCR79	R line
15	TCB114	B line	65	TCB168	B line	115	TCB48	B line	165	TCR110	R line	215	TCR172	R line	265	TCR8	R line
16	TCB115	B line	66	TCB169	B line	116	TCB49	B line	166	TCR111	R line	216	TCR18	R line	266	TCR80	R line
17	TCB116	B line	67	TCB170	B line	117	TCB5	B line	167	TCR112	R line	217	TCR19	R line	267	TCR81	R line
18	TCB117	B line	68	TCB171	B line	118	TCB51	B line	168	TCR113	R line	218	TCR2	R line	268	TCR82	R line
19	TCB119	B line	69	TCB172	B line	119	TCB56	B line	169	TCR114	R line	219	TCR20	R line	269	TCR83	R line
20	TCB12	B line	70	TCB173	B line	120	TCB57	B line	170	TCR115	R line	220	TCR21	R line	270	TCR84	R line
21	TCB120	B line	71	TCB174	B line	121	TCB59	B line	171	TCR116	R line	221	TCR22	R line	271	TCR85	R line
22	TCB121	B line	72	TCB175	B line	122	TCB60	B line	172	TCR117	R line	222	TCR23	R line	272	TCR86	R line
23	TCB122	B line	73	TCB176	B line	123	TCB63	B line	173	TCR118	R line	223	TCR24	R line	273	TCR87	R line
24	TCB123	B line	74	TCB177	B line	124	TCB66	B line	174	TCR119	R line	224	TCR25	R line	274	TCR88	R line
25	TCB124	B line	75	TCB178	B line	125	TCB67	B line	175	TCR12	R line	225	TCR26	R line	275	TCR9	R line
26	TCB125	B line	76	TCB179	B line	126	TCB68	B line	176	TCR120	R line	226	TCR27	R line	276	TCR90	R line
27	TCB127	B line	77	TCB18	B line	127	TCB72	B line	177	TCR121	R line	227	TCR28	R line	277	TCR92	R line

Table S3 Continued…

28	TCB128	B line	78	TCB180	B line	128	TCB73	B line	178	TCR122	R line	228	TCR29	R line	278	TCR93	R line
29	TCB129	B line	79	TCB181	B line	129	TCB74	B line	179	TCR123	R line	229	TCR3	R line	279	TCR94	R line
30	TCB13	B line	80	TCB182	B line	130	TCB75	B line	180	TCR125	R line	230	TCR30	R line	280	TCR95	R line
31	TCB130	B line	81	TCB183	B line	131	TCB77	B line	181	TCR128	R line	231	TCR31	R line	281	TCR99	R line
32	TCB131	B line	82	TCB184	B line	132	TCB78	B line	182	TCR129	R line	232	TCR32	R line			
33	TCB132	B line	83	TCB185	B line	133	TCB79	B line	183	TCR13	R line	233	TCR4	R line			
34	TCB134	B line	84	TCB186	B line	134	TCB8	B line	184	TCR130	R line	234	TCR42	R line			
35	TCB136	B line	85	TCB187	B line	135	TCB80	B line	185	TCR131	R line	235	TCR43	R line			
36	TCB138	B line	86	TCB188	B line	136	TCB81	B line	186	TCR132	R line	236	TCR44	R line			
37	TCB139	B line	87	TCB189	B line	137	TCB82	B line	187	TCR133	R line	237	TCR45	R line			
38	TCB140	B line	88	TCB190	B line	138	TCB83	B line	188	TCR134	R line	238	TCR49	R line			
39	TCB141	B line	89	TCB191	B line	139	TCB84	B line	189	TCR135	R line	239	TCR5	R line			
40	TCB142	B line	90	TCB192	B line	140	TCB85	B line	190	TCR136	R line	240	TCR50	R line			
41	TCB143	B line	91	TCB193	B line	141	TCB86	B line	191	TCR137	R line	241	TCR52	R line			
42	TCB144	B line	92	TCB2	B line	142	TCB87	B line	192	TCR138	R line	242	TCR53	R line			
43	TCB146	B line	93	TCB20	B line	143	TCB88	B line	193	TCR139	R line	243	TCR54	R line			
44	TCB147	B line	94	TCB21	B line	144	TCB89	B line	194	TCR14	R line	244	TCR55	R line			
45	TCB148	B line	95	TCB23	B line	145	TCB9	B line	195	TCR140	R line	245	TCR57	R line			
46	TCB149	B line	96	TCB24	B line	146	TCB90	B line	196	TCR141	R line	246	TCR58	R line			
47	TCB15	B line	97	TCB25	B line	147	TCB91	B line	197	TCR142	R line	247	TCR59	R line			
48	TCB150	B line	98	TCB26	B line	148	TCB92	B line	198	TCR144	R line	248	TCR6	R line			
49	TCB151	B line	99	TCB27	B line	149	TCB93	B line	199	TCR145	R line	249	TCR60	R line			
50	TCB152	B line	100	TCB28	B line	150	TCB94	B line	200	TCR15	R line	250	TCR61	R line			
